# Extensive epigenetic modification with large-scale chromosomal and plasmid recombination characterise the *Legionella longbeachae* serogroup 1 genome

**DOI:** 10.1038/s41598-022-09721-9

**Published:** 2022-04-06

**Authors:** Sandy Slow, Trevor Anderson, David R. Murdoch, Samuel Bloomfield, David Winter, Patrick J. Biggs

**Affiliations:** 1 Department of Pathology and Biomedical Science, University of Otago, Christchurch, New Zealand; 2 Microbiology, Canterbury Health Laboratories, Christchurch, New Zealand; 3 Molecular Epidemiology and Veterinary Public Health Laboratory, Infectious Disease Research Centre, School of Veterinary Science, Massey University, Palmerston North, New Zealand; 4 Bioinformatics and Statistics Group, School of Fundamental Sciences, Massey University, Palmerston North, New Zealand; 5 Present Address: Agricultural Sciences Department, Lincoln University, Canterbury, New Zealand; 6 Present Address: The Quadram Institute Bioscience, Norwich Research Park, Norwich, UK; 7 Present Address: Institute of Environmental Science and Research, Wellington, New Zealand

**Keywords:** Microbial genetics, Pathogens

## Abstract

*Legionella longbeachae* is an environmental bacterium that is the most clinically significant *Legionella* species in New Zealand (NZ), causing around two-thirds of all notified cases of Legionnaires’ disease. Here we report the sequencing and analysis of the geo-temporal genetic diversity of 54 *L. longbeachae* serogroup 1 (sg1) clinical isolates, derived from cases from around NZ over a 22-year period, including one complete genome and its associated methylome. The 54 sg1 isolates belonged to two main clades that last shared a common ancestor between 95 BCE and 1694 CE. There was diversity at the genome-structural level, with large-scale arrangements occurring in some regions of the chromosome and evidence of extensive chromosomal and plasmid recombination. This includes the presence of plasmids derived from recombination and horizontal gene transfer between various *Legionella* species, indicating there has been both intra- and inter-species gene flow. However, because similar plasmids were found among isolates within each clade, plasmid recombination events may pre-empt the emergence of new *L. longbeachae* strains. Our complete NZ reference genome consisted of a 4.1 Mb chromosome and a 108 kb plasmid. The genome was highly methylated with two known epigenetic modifications, m^4^C and m^6^A, occurring in particular sequence motifs within the genome.

## Introduction

In both natural and man-made environments *Legionella* spp. bacteria are ubiquitous intracellular parasites of protozoa. Humans are “accidental hosts” when lung macrophages become infected following exposure to contaminated materials, causing Legionnaires’ disease (LD), an often-severe pneumonia. In New Zealand (NZ), which has the highest reported incidence of LD in the world^1,2^, *Legionella longbeachae* is the species that causes nearly two-thirds of all notified cases^2–4^. Of the two serogroups, serogroup 1 (sg1) is the most clinically relevant. Unlike *L. pneumophila*, the predominant disease causing species in the UK, Europe and USA^1,5^, *L. longbeachae* is primarily found in soil and composted plant material^6,7^. As a result, most cases of LD in NZ occur during spring and summer when people at greatest risk are those involved in gardening^2,4,7–9^.

Although, whole genome sequencing and subsequent data interrogation provides invaluable insights into the biology, evolution and virulence of pathogenic organisms, until recently, genomic data for *L. longbeachae* was sparse, consisting of a single complete genome from an Australian sg1 isolate (NSW150) and four draft genomes; two sg1 and two sg2 isolates^10,11^. As such, comparative genomic analyses between *L. longbeachae* and other *Legionella* species was limited, with many using the complete NSW150 genome as the sole *L. longbeachae* representative^12,13^. Despite this, such analyses revealed a larger (≈ 500 kb) and differently organised chromosome than *L. pneumophila*^10–12,14^.

The relatively recent emergence of *L. longbeachae* as an important cause of LD in Europe and the UK^15^, particularly a 2013 outbreak in Scotland^16^, prompted concerted efforts in genomic sequencing, with a substantial increase in the amount of genomic data. A large-scale sequencing project of 64 clinical and environmental isolates was reported in 2017^17^. Analysis of this additional sequence data revealed further complexity, revealing variation is driven largely by extensive intra-species horizontal gene transfer and recombination. There is also evidence of inter-species gene transfer via plasmids that are the result of recombination between various plasmids of the different *Legionella* species^17^. Two more complete genomes have also been obtained, including for the ATCC type-strain from one of the first reported *L. longbeachae* LD cases in Long Beach, California in 1980 (GenBank: FDAARGOS_201^18^) and one we sequenced as part of this study from a NZ patient hospitalised with LD in 2014 (F1157CHC; GenBank NZ_CP020894^19^).

Although such genomic data has provided valuable information^10,17^, there is scope for further large-scale genomic sequencing to more fully assess genetic relationships, determine changes over time and define regions important for virulence and pathogenesis. Given the clinical significance of *L. longbeachae* in NZ, we have obtained the genome sequence of 54 sg1 clinical isolates, including one complete NZ reference genome^19^ and its associated epigenomic data. The isolates were derived from non-outbreak LD cases from 8 regions around NZ over a 22-year period (1993–2015), allowing us to examine its geo-temporal genetic diversity through ancestral state reconstruction and phylogenic analysis, chromosome and plasmid architecture and for the first time, characterise its epigenome.

## Results and discussion

### Geo-temporal and phylogenetic relationships of NZ sg1 isolates

**Figure 1:**
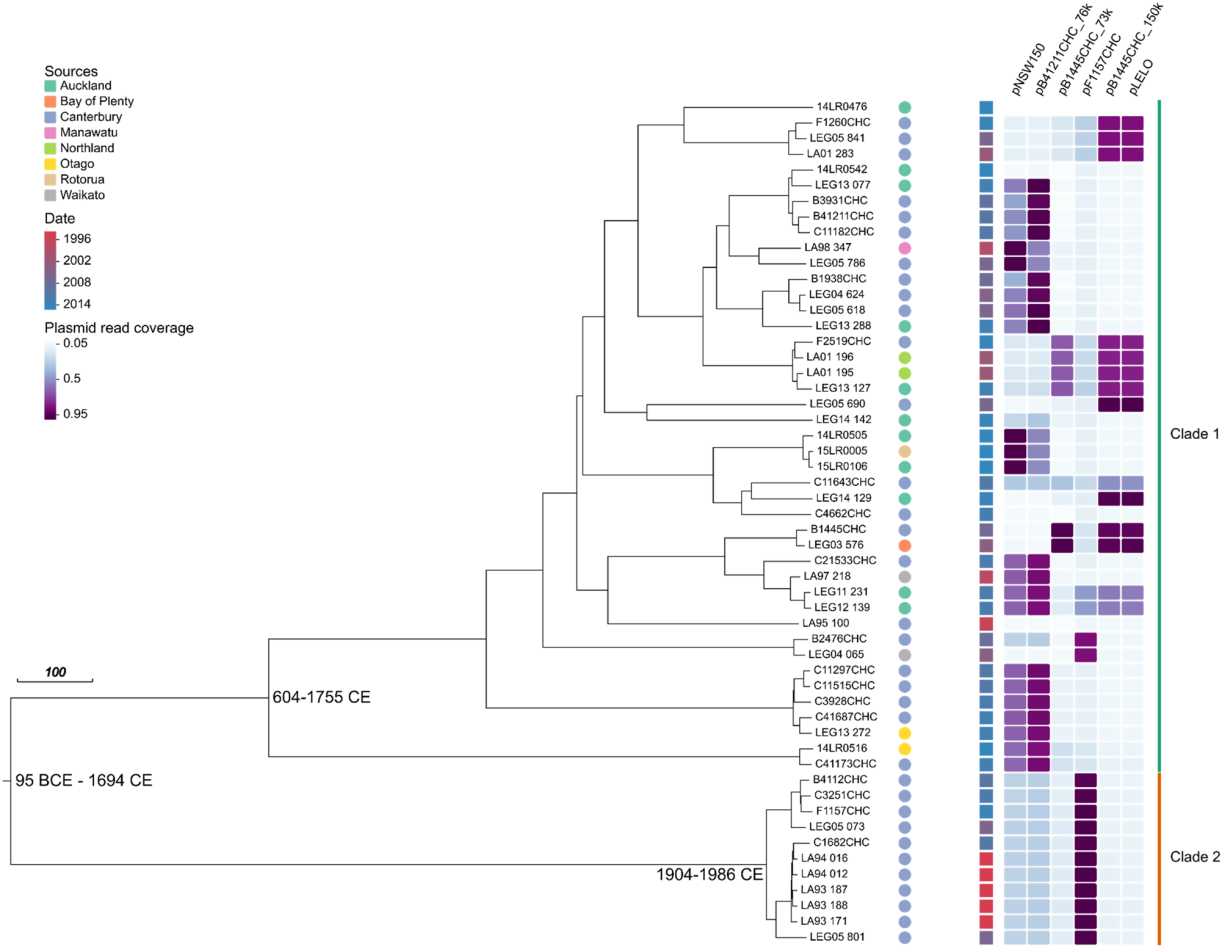
Maximum clade credibility tree of 54 *L. longbeachae* clinical isolates. The scale bar represents the length of 100 years. Isolates are coloured by date of collection (squares), region (circles) and plasmid read coverage (heat map). The years in parentheses represent the estimated timing of coalescent events (95% Highest Posterior Density interval).

The 54 *L. longbeachae* sg1 isolates used in our analysis were found to share 5383 core SNPs (2338 post Gubbins). Phylogenetic modelling estimated a substitution rate of 4.70 × 10^–8^–2.26 × 10^–7^ substitutions site^−1^ year^−1^ and shared a date of common ancestor between the years 95 BCE–1694 CE (95% HPD interval, Fig. 1). This substitution rate is similar to that of *L. pneumophila*^20^. There was extensive recombination, as evidenced by the large number of recombinant SNPs identified via Gubbins (3045) and the isolates belonged to two main clades (Fig. 1). The larger clade consisted of isolates from several regions in NZ and shared a date of common ancestor between the years 604–1755 CE (95% HPD interval), while the smaller clade consisted of isolates from the Canterbury district only, sharing a date of common ancestor between 1904 and 1986 CE (95% HPD interval; S1 Figure). Given the ad hoc nature of our sampling and the strong bias towards the Canterbury region, it is not clear if the smaller clade truly reflects the distributions of these lineages within NZ.

The oversampling of Canterbury isolates is primarily a historical consequence stemming from the 1990’s when pneumonia aetiological studies in this region revealed the importance of *Legionella* as a cause of pneumonia^21,22^. As a result, systematic routine testing, including a commitment to culture patient specimens allowing the isolation of clinical strains, was initiated, which subsequently lead to a disproportionate number of isolates being derived from the region. Recently it has been shown that when routine, systematic testing similar to that used in Canterbury is performed in other NZ regions, there are several that have higher or similar rates of LD^2^. Thus, the higher rates previously observed in Canterbury appear to predominantly reflect the testing regime employed rather than some special or intrinsic quality of the region. Future studies that include more isolates from outside Canterbury will clarify this and potentially identify pathways that has led to its introduction to NZ.

One potential explanation for the splitting of the two clades is that there have been two separate introductions into NZ. *L. longbeachae* has recently been detected by qPCR on the bark of live pine trees^23^, particularly on the species *Pinus radiata*, which is an important commercial crop and the most common pine tree species grown in NZ^24^. It was first introduced in the 1850’s, but the boom in commercial forestry did not begin until 1920–1930. This boom coincides with the date of the common ancestor of clade 2 and many sub-clades of clade 1. This suggests *L. longbeachae* may have been introduced to NZ with *Pinus radiata* followed by relatively rapid evolution and dispersal. The swift evolution and spread of disease-causing strains may be a feature of *Legionella*. It has also been observed in *L. pneumophila*, where David et al.^20^, showed phylogenetic evidence of rapid dispersion and the emergence of disease-causing strains in man-made environments over the last 100 years.

In our plasmid analyses, three additional *L. longbeachae* plasmids were identified through further sequencing of two of our clinical isolates, B1445CHC and B41211CHC. Isolate B1445CHC was found to contain two plasmids of 73,728 bp and 150,426 bp (pB1445CHC_73k and B1445CHC p150k), while B41211CHC contained only one plasmid of 76,153 bp (pB41211CHC_76k). Alignment of the NZ *L. longbeachae* read sets to the *Legionella* reference plasmids (pNSW150, pB41211 CHC_76k, pB1445CHC_73k, pF1157CHC, pB1455CHC_150k and pLELO) demonstrated that some of the NZ isolates contained an exact copy of the reference plasmids investigated, some contained similar plasmids with reads aligning to sections of the reference plasmids, and some contained reads that aligned to sections of more than one reference plasmid (Fig. 1). This illustrates that the *Legionella* plasmids share a common back bone separated by variable regions and that there is extensive recombination amongst them (S2 Figure). The plasmid results also correlated with the clades (Fig. 1) identified via phylogenetic analysis, suggesting that plasmid recombination events may pre-empt the emergence of new *L. longbeachae* strains.

Our global analysis that included available sequence information from 89 *L. longbeachae* isolates from the United Kingdom (UK) and NZ were found to share 3219 core SNPs and belonged to multiple small clades. Most of the clades consisted of isolates from a single country, whilst a small number had isolates from both countries (S3 Figure), indicating some recent global transmission.

### Genetic diversity of *L. longbeachae* sg1 clinical isolates

**Figure 2:**
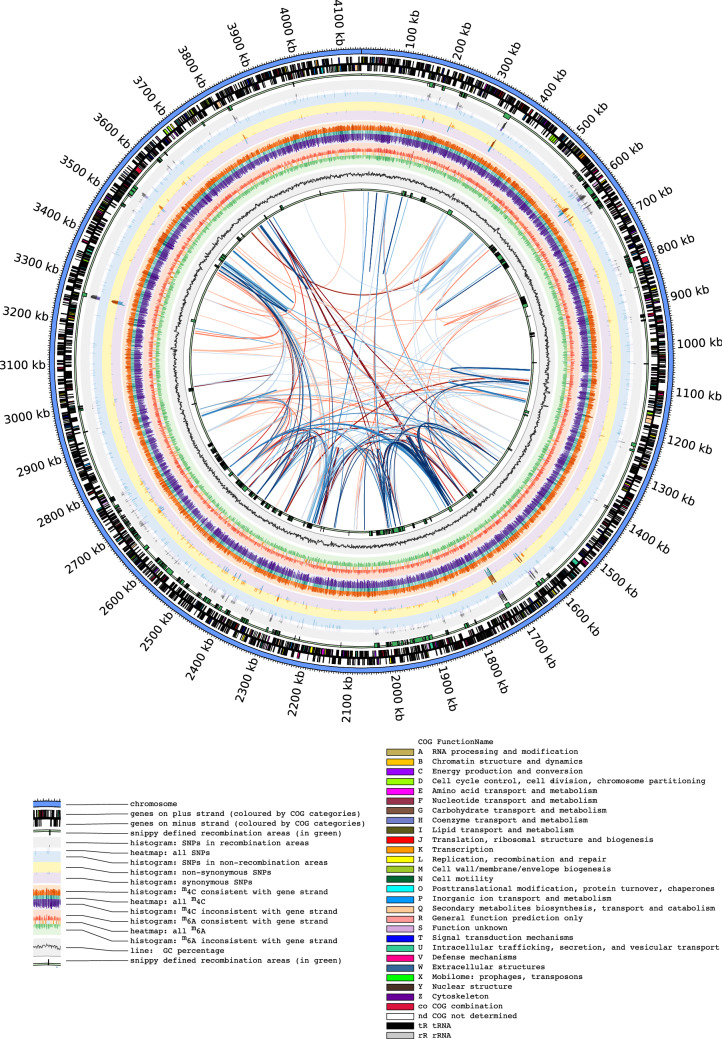
Circos plot of NZ *L. longbeachae* isolate F1157CHC. Tracks from the outside to the inside and the predicted gene functional COG category colours are denoted in the key. All data for the SNPs, methylation patterns and GC percentage are values calculated in non-overlapping 1 kb bins. The data for the SNPs and methylation patterns are shown in a log10 scale, and the histograms are also coloured so that larger values are in darker colours. In the centre of the plot are the results from the Reputer analysis, with palindromic repeats in red and forward repeats in blue. The repeats are darker in colour with a smaller Hamming distance between the repeats.

Given there are few complete *L. longbeachae* genomes available we chose one of our sg1 isolates as the reference genome to further analyse our other 53 NZ isolates. Isolate F1157CHC was sequenced using both Illumina short read sequencing in the initial comparative dataset, and then it was subsequently sequenced with PacBio long read sequencing. To visualise the data from the comparison of the 54 samples in the dataset and to show multiple facets of this study simultaneously, an overarching Circos figure (Fig. 2) was generated using the complete PacBio genome of F1157CHC as a backbone. The tracks in the figure are described in detail in the legend. Overall it can be seen that the regions detected for recombination by Gubbins are unevenly distributed, with some clusters around the genome (~ 600 kb, ~ 800 kb, ~ 1900–2050 kb), and a large, slightly less dense region (2300–2800 kb). In total, 655 protein coding genes are at least partially included in these regions of high recombination, and they are not clearly associated with any functional class (S4 Figure).

As indicated in the gene rings in Fig. 2, the genome of F1157CHC was functionally annotated and categorized using the amino acid sequences from the NCBI PGAP predictions against the eggNOG-mapper database (v. 2.0). The genes were coloured according to their COG categories (Fig. 2). We found that 3410 (94.14%) returned an annotation result, and of those 2741 (80.38%) were categorized with COG functional categories (S1 Table). In terms of the performance of the eggNOG server, this level of annotation for *L. longbeachae* is slightly above the level of 76% reported for the Legionellales order, and close to the eggNOG v. 5.0 database average of 80%^25^. The main functional groups (those with a single COG category definition) accounted for 2522 (73.96%) of the annotations. As expected, the functions of the genes encoded on the chromosome and plasmid differ (S5 Figure), where it carries more genes of unknown function (category S) and those associated with replication, recombination and repair (category L). COG category S—“function unknown”—is the largest single category, and accounts for 547 (16.04%) of the returned annotations.

We used our set of 53 draft genomes to investigate both the core and pangenome. A genome summary of these 53 draft genomes, plus the complete genomes of F1157CHC, NSW150 and FDAARGOS_201, can be found in S2 Table. All 53 draft genomes have a similar length (4,148,006.3 bp ± 78,321.3), GC content (37.11% ± 0.05), coding sequences (3576.2 ± 81.8), signal peptides (277.6 ± 7.8) and tRNAs (45.1 ± 3.0). The complete genome sequences are on average longer (4,194,301.3 bp ± 66,793.1) and less GC-rich (37.15% ± 0.10) than the draft genomes, and also contain more protein coding genes (3609.7 ± 76.8), tRNA’s (48.3 ± 3.2), and proteins with signal peptides (282.7 ± 5.5). These differences are small and not significant, suggesting genome-completeness doesn’t strongly influence our ability to annotate genomes. The only exception is the number of rRNAs, which are much more numerous in the complete genomes (12 copies each) than the draft genomes (6.89 ± 0.42), reflecting the difficulty in assembling highly repetitive regions from short read sequencing data.

In comparison to the recent study of Bacigalupe et al.^17^, (n = 56, predominantly UK sg1 isolates), we found that the range of our coding sequences was similar to the 3558 genes they reported. We cannot say if there is any real difference in gene numbers between the NZ strains reported here, and those in the Bacigalupe et al., study^17^ because only summary gene numbers per genome were provided, but it seems unlikely.

Using Roary^26^, we found a pangenome of 6517 genes, and a core genome of 2952 genes amongst 56 isolates (our 54 isolates, NSW150 and FDAARGOS). This is ~ 86.3% of the number of genes in the F1157CHC genome, indicating a large core genome and a small accessory genome amongst the isolates in this study. Bacigalupe et al.^17^, also reported a core genome (2574 genes) and pangenome (6890 genes), which were over a shorter, but contemporaneous, timeframe. Given the isolate numbers are almost the same (excluding reference isolates), but the methodologies differ for calculating the core genome, it is interesting to observe a smaller number of genes in the core but a larger number of genes in the pangenome. It is tempting to speculate that there might be a smaller gene repertoire for *L. longbeachae* in NZ, possibly a result of its relative geographical isolation, or maybe environmental conditions are different, requiring the use of different sets of genes to survive within NZ soil. Using the categories defined within Roary, we found 157 genes (95 to 99% of strains) in the soft core category, 865 (15 to 95%) in the shell category and 2543 (0 to 15%) in the cloud category (S1 File).

Currently, there are 61 recognised species and 3 subspecies within the *Legionella* genus (http://www.bacterio.net/legionella.html). Of these, 58 have at least draft genome sequences available, which aid in understanding the evolution of the genus^27^. A core genome has been estimated to be only 1008 genes, highlighting genus diversity. With a GC content of ~ 39% and a genome of ~ 3.3 Mb, *L. pneumophila* is regarded as the most clinically important^1^. A recent Australian study^28^ has estimated the core genome of this species as 2181 genes, which is 36.7% of the pangenome’s genes (5919 genes). In comparison, in our study, analogous numbers indicate 45.3% for *L. longbeachae*, suggesting its genome is probably more stable than the *L. pneumophila* genome.

Finally, we used FastGeP^29^ to perform an ad-hoc whole genome MLST analysis of the 56 isolates using the 3420 CDSs in the F1157CHC reference genome. We found 2756 loci were shared, of which 1321 (47.93%) were identical at the allelic level. One-hundred and eight of the shared loci were excluded because of hypothetical gene duplications, and 664 were excluded because of incomplete information, such as missing alleles, truncation or nucleotide ambiguities. After removal of these loci, 2648 (of which 1327 were polymorphic) were used to construct the distance/difference matrix. As FastGeP is looking at allelic distances in a gene, the distance between two alleles is independent of underlying sequence differences. Visualization of the FastGeP matrix in iTOL^30^ is shown in S2 file.

### Antibiotic resistance and virulence factor genes

Unsurprisingly, our 54 *L. longbeachae* sg1 isolates all contained a chromosomal class D β-Lactamase gene homologous to *bla*_*OXA*_ enzyme family. This 795 bp *bla*_*OXA-like*_ gene, whose phenotypic features are uncharacterized, is also found in *L. oakridgensis* (100% nucleotide match). Twenty-one isolates also have another molecular class D β-Lactamase with 100% nucleotide match to *bla*_*OXA-*29_ that are contained on a plasmid similar to *L. pneumophila* pLELO. The *bla*_*OXA-*29_ gene was first identified in the *Fluoribacter gormanii* type strain ATCC 33297^T^ (Genbank accession number NG_049586.1^31^). The majority of the known class D β-Lactamases are found on mobile genetic elements and indicate the intra-species transfer of *bla*_*OXA-*29_ on conjugative plasmids amongst the various *Legionella* species such as *L. pneumophila*, *L. sainthelensi*, and *L. hackeliae*. This *bla*_*OXA-*29_ β-Lactamase is part of a group of structurally related serine enzymes that have high activity against penicillins, reduced activity against cephalosporins and no activity against carbapenems^32^.

All isolates also contained a previously identified tetracycline destructase gene, *tet56* that confers tetracycline resistance when expressed^33^. Tet56 belongs to a family of flavoprotein monoogenoxygenases that inactivate tetracycline antibiotics through covalent modification to the antibiotic scaffold^33,34^. Previously, the antimicrobial susceptibilities of 16 isolates that were sequenced in our current study had been investigated^35^. For these isolates, the tetracycline MIC_90_ was found to be high, ranging between 16 to 64 mg/mL when the isolates were grown in BYE broth, suggesting *tet56* was expressed and the protein was functional in these isolates (S3 Table).

Virulence factor database analysis showed our 54 isolates as well as the NSW150 and FDAARGOS_201 complete genomes had a near identical pattern with between 33 and 36 virulence factor genes (S4 Table). Many of these encoded various components of the type IVB Dot/Icm secretion system (T4SS), which is essential for its virulence and found to be present in all *Legionella* species examined to date^36^.

### *Legionella longbeachae* chromosome and plasmid architecture

Our complete chromosome for isolate F1157CHC has been published^19^, and therefore the description of this genome is kept relatively brief and is more comparative in nature with the other available reference *L. longbeachae* genomes (NSW150, and FDAARGOS_201).

**Figure 3:**
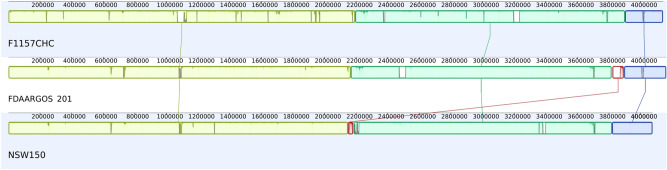
Mauve alignment of the three complete *L. longbeachae* sg1 genomes, F1157CHC, FDAARGOS and NSW150 from top to bottom. The 4 main collinear blocks are indicated by colours (LCB1 is red, LCB2 is yellow, LCB3 is green and LCB4 is blue). The sizes of the blocks for LCB1, LCB2, LCB3 and LCB4 are ~ 81 kb, ~ 2265 kb, ~ 1807 kb and ~ 272 kb respectively. Lines between LCBs in the isolates are for the same LCB. In the visualisation the areas within the LCBs without colour indicates underlying differences in the LCB, as explained by the fact that the LCBs are different sizes in each isolate.

We compared all three reference genomes using the MAUVE plugin within Geneious (v 9.1.8) and the results are shown in Fig. 3. At 4,142,881 bp, F1157CHC is larger by 65,549 bp when compared to NSW150 and smaller by 19,851 bp to FDAARGOS_201. Overall, the genomes of F1157CHC, NSW150 and FDAARGOS_201 are similar in their organisation with the MAUVE alignment showing four (81–2264 kb) collinear blocks in the genomes, called LCB1, LCB2, LCB3 and LCB4. At an overall genome level, the order and orientation of these blocks indicates a greater similarity between NSW150 and F1157CHC, while FDAARGOS_201 is slightly different (S5 Table).

Three of these blocks (LCB2, LCB3 and LCB4) are found in all three genomes, and a further one (LCB1) is found only in NSW150 and FDAARGOS_201. The genomic coordinates and the percentage of the collinear block that contains genomic sequence are described in S5 Table. In addition, there are two and three small regions in two of the genomes that are not found in collinear blocks totaling 4.2 and 4.4 kb for NSW150 and FDAARGOS_201, respectively. For FDAARGOS_201 and NSW150, two of these unique regions are found flanking the shortest collinear block of 81 kb (LCB1), and for NSW150 the third region is a short sequence at the start of the chromosome (unusually for this chromosome the start of the *dnaA* gene is not annotated to be at position 1). The LCB1 block shows the greatest disparity in content with the genomic length in NSW150 being 31.3 kb, but 73.6 kb in FDAARGOS_201, hence there are many gaps in the collinear block alignments.

It should be noted that as the MAUVE aligner within Generous works on a linear chromosome, the LCBs at the end of the chromosome form part of the same larger collinear block, meaning that on the circular chromosomes there are in effect only three blocks, with the ~ 1807 kb block LCB3 being flanked by the content variable 81 kb block LCB1. There are thus only a few boundaries around the main collinear blocks. The boundary between LCB2 and LCB3 in FDAARGOS_201 and FH1157CHC occurs within the traF gene, part of the *tra* operon. The organization is more complex in NSW150 where the 31.5 kb block of LCB1 and a 3.9 kb region containing three hypothetical genes is found between LCB2 and LCB3, with the *tra* operon being found on LCB1. The *tra* operon is important for pathogenicity because it forms part of the T4SS for the transfer of plasmids via conjugation^37^. The other main boundary between LCB3 and LCB4 for all three chromosomes, the transfer messenger RNA (tmRNA) *ssrA* gene is present at the end of LCB4. The tmRNA genes are part of the trans-translocation machinery, which can overcome ribosome stalling during protein biosynthesis. Trans-translocation has been found to be essential for viability within the *Legionella* genus, with the *ssrA* gene being unable to be deleted in *L. pneumophila*^38^. Under the control of an inducible promoter, it was found that decreasing tmRNA levels led to significantly higher sensitivity to ribosome-targeting antibiotics, including erythromycin^38^. At the end of LCB3 in F1157CHC and NSW150, there is an IS6 family transposase and an SOS response-associated peptidase, but little is known about these genes. The flanking gene in FDAARGOS_201 comes from a small orphan block of 1.3 kb between LCB1 and is a short DUF3892 domain-containing protein, as defined by Pfam^39^. Whilst having unknown function it is found widely across bacteria and archaea, and within the Legionellales order.

As described above, the collinear blocks include gaps, and except for LCB1, all other defined blocks in the isolates are found with the genomic length being greater than 87% of the block length. Within the blocks themselves, LCB1 shares a common region of ~ 23.8 kb and a larger non-overlapping (i.e. different gene content) region in FDAARGOS_201 compared to NSW150. For the remaining three blocks there are combinations of absence and presence of genetic material within these blocks across the three isolates. For the regions over 10 kb, these can be summarized as regions that are present in only a single isolate (37.1 kb in LCB3 of F1157CHC), or in two isolates (12.0 kb in LCB2 of FDAARGOS_201 and NSW150), and those that are different across all three isolates (86.3 kb in LCB2, 12.2 kb in LCB2 and 18.1 kb in LCB3). Other more complex combinations account for a further six regions of the blocks involving three regions for F1157CHC and NSW150 (19.3 kb in LCB2 with a differing FDAARGOS_201 sequence; 64.4 kb in LCB3 with an extra sequence in NSW150; and 43.4 kb in LCB3, a large FDAARGOS_201 sequence), two for FDAARGOS_201 & NSW150 (40.0 kb in LCB4 with different flanking sequence for FDAARGOS_201 and F1157CHC; and 11.3 kb in LCB2 with a different sequence for F1157CHC), and one for FDAARGOS_201 & F1157CHC (101.3 kb in LCB3 with a different sequence for NSW150). The gene content in these blocks is varied, and the boundaries close to tRNA genes, site-specific integrase genes, SDR family oxidoreductase genes, ankyrin repeat domain-containing genes, or in intragenic space, but for some of the boundaries transposase genes (IS3, IS4, IS6, and IS926 families) are involved. In bacteria, tRNAs have been shown to be integration sites^40^, so finding them at collinear block boundaries is unsurprising.

**Figure 4:**
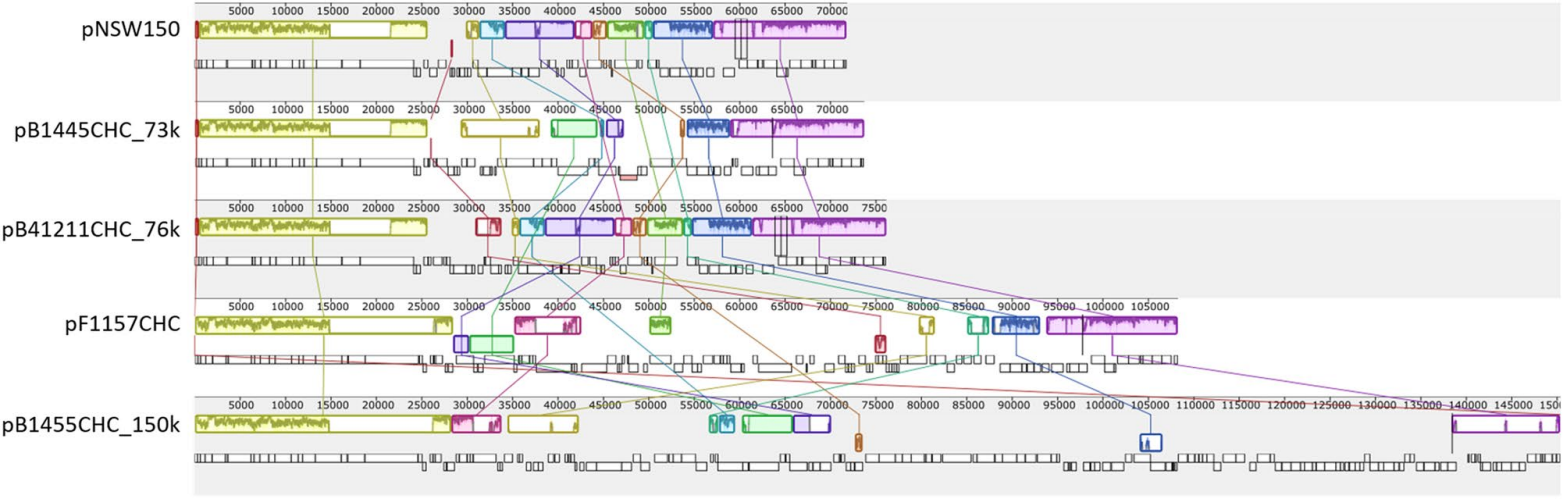
Mauve alignment of five *L. longbeachae* reference plasmids.

Only NSW150 and F1157CHC were found to contain a plasmid (pNSW150 and; pF1157CHC^19^). At 108,267 bp pF1157CHC is 36,441 bp larger when compared to pNSW150. To assess plasmid architecture more fully, the three additional *L. longbeachae* plasmids we obtained from further sequencing of two of our isolates (pB1445CHC_73k, pB1445CHC_150k and pB41211CHC_76k) were aligned using MAUVE and visualised in Geneious, (Fig. 4). The plasmids share a common backbone consisting of conjugational genes (yellow collinear block), ranging in size from ~ 25,000 to ~ 28,000 bp, as well as several other collinear blocks that vary in size and orientation (Fig. 4). These blocks are separated by variable regions around mobile genetic elements, such as insertion sequences. Analysis of the larger plasmid pB1445CHC_150k revealed this is the same as plasmid pLELO, first reported in *L. pneumophila*. MAUVE alignment of the *L. longbeachae* plasmids, pLELO and two *L. sainthelensi* plasmids (pLA01-117_165k and pLA01-117_122k^41^) (S6 Figure) again shows *Legionella* plasmids have a common backbone including conjugational genes separated by variable regions.

Although the number of plasmids in our analysis is limited, *Legionella* plasmids identified to date can be broadly divided into two groups; one consisting of the smaller plasmids of ~ 70 kb that appear to be primarily a *L. longbeachae* group (pNSW150, pB1445CHC_73k, pB41211CHC_76k) and another group consisting of larger plasmids that occur in various species, including our complete genome (pF1157CHC, pLELO, pLA01-117_165k). This again suggests there has been extensive plasmid recombination followed by both intra- and inter-species transfer, supporting the findings of Bacigalupe et al.^17^.

**Figure 5:**
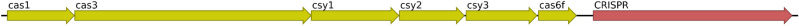
The type I-F CRISPR-Cas element found in some of the *L. longbeachae* isolates sequenced in this study.

Interestingly, pB1445CHC_73 k has a repetitive region that was identified as a clustered regularly interspaced short palindromic repeat (CRISPR) element. This element belongs to the type I–F system with the same repeat region between 20 to 33 spacer regions and cas1-cas6f associated enzyme (Fig. 5). While there are few reports of naturally occurring CRISPR-Cas arrays on plasmids, previous studies^42,43^ as well as a recent comparative genomics analysis of available bacterial and archaeal genomes has demonstrated that type IV CRISPR-Cas systems are primarily encoded by plasmids^44^. There have also been similar reports of a type I-F CRISPR-Cas array being present on the plasmids of *L. pneumophila* strains^45,46^. Further analysis of our other *L. longbeachae* isolates showed that the type I–F CRISPR-Cas element is also present in 5 other strains (F2519CHC, LA01-195, LA01-196, LA03-576).

### *Legionella longbeachae* methylome

**Figure 6:**
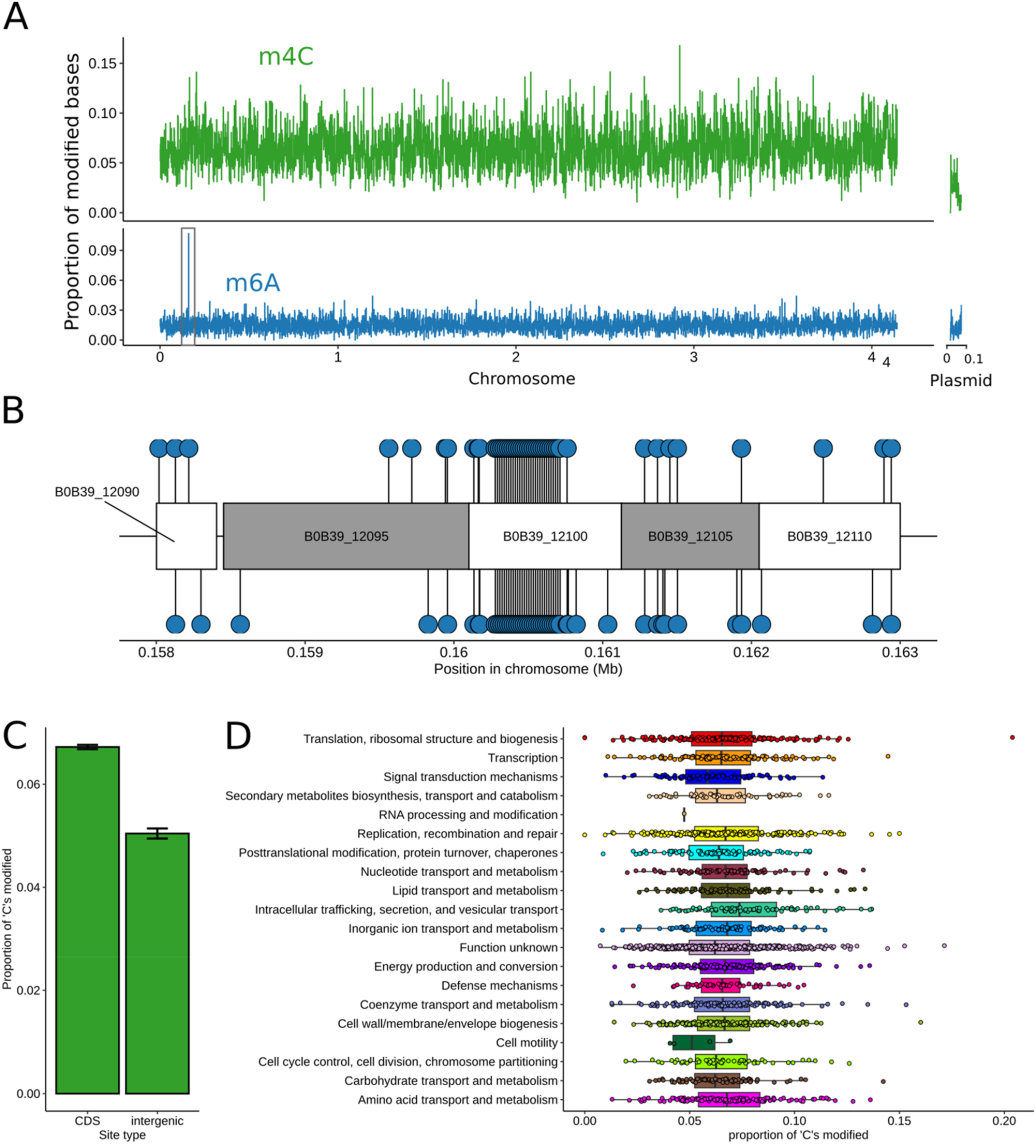
Methyl-distribution and methyl COG. Both methylation marks are approximately evenly distributed across the *L. longbeachae* genome. (**A**) The frequency with which m^4^C (above in green) and m^6^A (below in blue) modifications were detected is plotted in 1 kb windows, note the difference in y-axes for each sub-plot. The black box represents the region of unusually high m^6^A modifications highlighted in (**B**) and the locations of individual m^6^A modifications are shown as blue circles for the region with an unusually high rate of this mark. The alternating white and grey boxes represent genes in this region, and are labelled with their NCBI locus ID. The gene responsible for the very high rate of m^6^A modification, B0B39_12100, is a tetratricopeptide repeat protein. m^4^C methylation is not strongly associated with any functional class. (**C**) The proportion of ‘C’ nucleotides are with evidence for methylation in coding and intergenic sequences. Error bars represent a 95% confidence interval, calculated using the normal approximation of a binomial distribution. (**D**) Each point represents the proportion of ‘C’ nucleotides in a given gene that show evidence for methylation. The genes are grouped and shaded by the COG category (x-axis). The box plots summarise the distribution of this value across each COG category.

The PacBio assay utilized in the current study is unable to detect 5mC modifications. However, methylome analysis of our F1157CHC genome identified two classes of modified base, N4-cytosine (m^4^C) and N6-methyladenine (m^6^A). Bases in the chromosomal sequence were more likely to be modified (1.49% of As and 6.4% of Cs being methylated) than those in the plasmid (1% of As and 2.4% of Cs) (Fig. 6A). Modifications were evenly distributed within a given molecule, except for a single cluster of m^6^A in the chromosome, where this methylation ‘spike’ is focused on a specific gene, BOB39_12100 as depicted in Fig. 6B. The majority (73.6%) of m^6^A bases occurred in three sequence motifs (ATGNNNNNNRTGG/CCAYNNNNNNCAT, GATC and GGGAG). Two of these (ATGNNNNNNRTGG/CCAYNNNNNNCAT and GATC) are almost always methylated (97–99.5% of occurrences) while the third (GGGAG) is frequently modified (77.2% of occurrences). By contrast, the m^4^C modifications are not strongly concentrated in motifs. The motif most frequently associated with this modification (CSNNNTB) is only modified in 9.2% of occurrences (about 3 times the background rate for all cytosines).

DNA methylation in bacteria is often associated with restriction modifications (RM) systems, which protect the bacterial cell from foreign DNA. These systems combine a restriction endonuclease that digests un-methylated copies of target sequences and a DNA methyltransferase that methylates this sequence motif in the bacterium’s own DNA. The strong association between m^6^A modification and three sequence motifs in the *L. longbeachae* genome suggests this modification is part of an RM system.

Using REBASE, we identified putative methyltransferases and encodnucleases in the *L. longbeachae* genome. This analysis revealed three neighbouring genes that encode a type I RM system associated with the ATGNNNNNNRTGG/CCAYNNNNNNCAT motif. Specifically, gene B0B39_08545 encodes a SAM-dependent DNA methyltransferase with target recognition domains for both ends of this motif, while genes B0B39_08550 and B0B39_08555 encode the S and R subunits of an associated endonuclease. The enzymes responsible for the GATC and GGGAG motifs are less clear. Two proteins (LloF1157ORF6795P and LloF1157ORF8795P) are homologous to methyltransferases that recognize GATC in other species. Neither of these proteins are associated with a restriction endonuclease.

Although many bacterial genomes contain the m^4^C modification, the biological functions encoded by it remains unclear^47^. There is some evidence that this mark may contribute to the regulation of gene expression. Notably, the deletion of a single m^4^C methyltransferase in *Helicobacter pylori* alters the expression of more than 100 genes and leads to reduced virulence. We used our genome annotation and methylation data to test for any associations between m^4^C methylation and genome features of functional classes of genes that might suggest this mark contributes to gene regulation in *L. longbeachae*. We found it is considerably more common within protein coding genes than intergenic spaces (Fig. 6C). However, there is no association between the presence of this mark in a gene sequence and any of the functional classifications present in our COG data (Fig. 6D). Although the over-representation of m^4^C bases in genetic sequences suggests it might be associated with, or a passive consequence of, transcription in *L. longbeachae*, we find no evidence it contributes to particular biological functions.

In summary, we have demonstrated that most genomic variability in *L. longbeachae* is from recombination with large-scale rearrangements in the chromosome. Our 54 sg1 clinical isolates could be grouped into two highly related clades that persisted over time. The most genetically distinct clade consisted of isolates from only the Canterbury region but could just reflect oversampling from this region. Further sequencing of isolates from other regions is required. Most sequenced isolates were found to contain a plasmid that showed high levels of recombination and horizontal gene transfer with evidence for both intra- and inter-species gene-flow. The genome of *L. longbeachae* was also highly modified, with m^6^A modifications being the most common and was strongly associated with particular sequence motifs.

## Materials and methods

### Bacterial isolates, sequencing and genome assembly

A total of 60 isolates previously identified as *L. longbeachae* (including 57 serotyped as sg1 and 3 serotyped either as sg2 or undefined) were sequenced. Isolates were obtained from either the NZ *Legionella* Reference Laboratory (ESR, Porirua, New Zealand; n = 39) or Canterbury Health Laboratories (CHL) culture collection (Christchurch, New Zealand; n = 21). All isolates were derived from sporadic LD cases that occurred between 1993 and 2015 from 8 regions (S7 Figure) around the country and included the first NZ case in which *L. longbeachae* was successfully cultured from a patient specimen (LA93_171; S2 Table).

Isolates were grown on buffered-charcoal-yeast-extract (BCYE) agar at 37 °C for 72 h. DNA was extracted from each fresh culture using GenElute Bacterial Genomic kits (Sigma-Aldrich, MO, USA) according to the manufacturer’s instructions. Libraries were prepared using the Nextera XT kit (Illumina, San Diego, CA, USA) and were sequenced using Illumina MiSeq technology (2 × 250 bp paired-end) and version 2 chemistry by NZ Genomics Ltd (University of Otago, Dunedin, NZ). The quality of the raw reads was checked using FastQC (v. 0.11.4; https://www.bioinformatics.babraham.ac.uk/projects/fastqc/). They were mapped against PhiX using Bowtie2 (v. 2.0.2^48^), and any that mapped to PhiX were removed from the SAM file, and read pairs were reconstructed using the SamToFastq.jar program from the Picard suite (v. 1.107; https://broadinstitute.github.io/picard/) using the default parameters. Any adaptors were removed through the “fastq-mcf” program (using the default parameters) from the ea-utils suite of tools (v. 1.1.2–621; https://expressionanalysis.github.io/ea-utils/). Finally the reads were quality trimmed using SolexaQA++ (v. 3.1.4^49^) at a probability threshold of 0.01 and sorted on length to remove any sequences < 50 bp prior to assembly. Sequence reads from each isolate was assembled using SPAdes (v. 1.2^50^) de novo assembler in “careful” mode, with default settings.

### Sequenced strains analysed

Of the 60 isolates (S2 Table), 57 were found to be *L. longbeachae* sg1, two were sg2 and one had been mistyped and was *Legionella sainthelensi*. Analyses were limited to the sg1 isolates but because poor sequence data were obtained for three genomes (2 from Auckland and 1 from Waikato), only 54 were included (Table 1). We also included the two other publicly available complete genomes for *L. longbeachae* sg1, NSW150 (Australia; GenBank: NC_013861) and FDAARGOS_201 (USA; GenBank: NZ_CP020412) in our core genome and cluster of orthologous groups (COG) analyses.

**Table 1:** Number of isolates sequenced and analysed by year group and region.

Region	Year group					Total
	1993–1997	1998–2002	2003–2007	2008–2012	2013–2015	
Northland	–	2	–	–	–	2
Auckland	–	–	–	2	9	11
Waikato	1	–	1	–	–	2
Bay of plenty	–	–	1	–	–	1
Rotorua	–	–	–	–	1	1
Manawatu	–	1	–	–	–	1
Canterbury	6	1	9	14	4	34
Otago	–	–	–	–	2	2
Total	7	4	11	16	16	54

The reads of a further 65 previously published *L. longbeachae* isolates (Bioproject number PRJEB14754, SRA accession numbers ERS1345649 to ERS1345585^17^) were downloaded and compared with our 54 sg1 isolates. However, 30 of these read sets were either of poor quality, aligning to less than 80% of our reference genome (F1157CHC; GenBank NZ_CP020894^19^), or were not *L. longbeachae* sg1 isolates and were excluded. The remaining 35 read sets were included in our global phylogenetic analyses (S6 Table).

### Ancestral state reconstruction and phylogenetic analysis

Single nucleotide polymorphisms (SNPs) were identified using Snippy v2.6 (https://github.com/tseeman/snippy). Snippy uses the Burrows-Wheelers Aligner^51^ and SAMtools^52^ to align reads of the 53 NZ *L. longbeachae* isolates to our reference genome *Legionella longbeachae* F1157CHC and FreeBayes^53^ to identify variants among the alignments. Gubbins was used to remove areas of recombination on the full alignment^54^. SNPs were exported into BEAUti v2.5 to create an Extensive Markup Language (xml) file for BEAST v2.5^55^. The *Legionella longbeachae* reference genome consists of 1,306,681 adenine, 765,717 cytosine, 772,189 guanine and 1,298,289 thymine. These nucleotides were added as constant sites to keep the model representative of *L. longbeachae*. bModelTest^56^ was used to choose the substitution model. Multiple molecular clock and tree models were trialed. Nested sampling (NS)^57^ was used to select the model (S3 File)^58^. A 121,324 Generalised Time-Reversible (GTR)^59^ model was used to model nucleotide substitutions, a constant coalescent model was used to model the effective population size, and an uncorrelated relaxed clock^60^ was used to model the molecular clock and was calibrated by tip dates. In the analyses, the substitution rate was restricted so it would not exceed 1 × 10^–4^ substitutions site^−1^ year^−1^ nor fall below 1 × 10^–8^ substitutions site^−1^ year^−1^^61^. Due to the large number of constant sites added, the proportion invariant was assumed to be zero. The .xml file was run in BEAST for 100 million steps, three times with different starting seeds, before LogCombiner was used to combine the runs with a 10% burn-in. Tracer v1.6 (Rambaut et al., http://beast.bio.ed.ac.uk/Tracer) was used to visualise the results. TreeAnnotator v2.6 was used to form a maximum clade credibility tree from the trees produced using BEAST. Evolview v2^62^ was used to visualise and edit the tree.

### Global *L. longbeachae* isolates

As described above the read sets of 35 previously published *L. longbeachae* isolates^17^ were downloaded and compared with our 54 NZ isolates using the SNP-identification method described above. In total, 89 *L. longbeachae* isolates from NZ and the UK were investigated for our global analysis. RaxML^63^ was used to form a maximum likelihood tree of the isolates based on their SNP data and was visualised using EvolView v2.

### Core genome and COG analyses

The eggNOG-mapper^25,64^) webserver with default parameters (http://eggnog-mapper.embl.de/) was used to annotate the F1157CHC PGAP-derived amino acid sequences. The Prokka pipeline (v. 1.12^65^) was used to annotate our draft isolates using default parameters. The Prokka-generated GFF files were analysed with Roary using default parameters, and the comparison script roary_plots.py was used to visualize the output. FastGeP was used with default parameters to perform a whole genome MLST analysis of the 56 isolates, which meant that the generated allele sequences were searched with BLAST + at an identity threshold ≥ 80%. F1157CHC was used as the reference genome for this analysis. SplitsTree (v.4.15.2^66,67^) was used to convert the FastGeP Nexus file into a Newick file (as a Neighbour-joining tree) for visualization and annotation in iTOL with the inclusion of metadata for region and the sample type.

### Antimicrobial resistance and virulence genes

Isolate contigs were screened using ABRIcate (v 0.8; https://github.com/tseemann/abricate and included plasmid finder, ncbi, card, vfdb, resfinder, argannot, echo and ecol_vf databases) to identify acquired resistance and virulence genes.

### Plasmid analyses

The contig reads of the 54 NZ *L. longbeachae* sg 1 isolates were aligned to five *L. longbeachae* reference plasmids pB1445CHC_73k, pB1445CHC_150k, pB41211CHC_76k, pF1157CHC and pNSW150 (Genbank: CP045304, CP045305, CP045307, CP020895 and NC_014544, respectively) using the Burrows-Wheeler aligner^51^. For each read set, the proportion of the plasmid with a read depth of ten or higher was calculated using samtools v1.9^52^. The plasmid sequences were annotated using Prokka v1.14.4^65^ and a dendrogram of gene presence-absence was formed using Roary v3.11.2^26^, before the sequences were aligned using EasyFig v2.2.4^68^.

To assess inter-species variation, the available sequences of one *L. pneumophila* plasmid pLELO (Genbank: NC_018141) and two *L. sainthelensi* plasmids pLA01-117_165k and pLA01-117_122k (Genbank: CP025492 and CP025493, respectively) with the five *L. longbeachae* reference plasmids were aligned and visualized using the MAUVE plugin within Geneious (v. 9.1.8).

### Complete NZ reference genome, gene prediction and annotation

To generate our own complete NZ reference genome, one isolate (F1157CHC) was further sequenced using the PacBio RSII system (Pacific Biosciences, CA, USA) as previously described^19^. Gene prediction and annotation was performed using the NCBI Prokaryotic Genome Annotation Pipeline (2013).

### Genome architecture

In order to assess the genome architecture, F1157CHC was used as the basis for all analyses in which comparisons were made against a reference. The genome was visualized using Circos software (v. 0.69.3^69^). Tracks included mapping the annotation prediction from PGAP, as well an overlay of the results of a functional annotation with the eggNOG web annotation server, mapping of both the methylation results and recombinant regions detected with Gubbins, SNP density of the comparative samples as defined by SNIPPY, and finally a visualization of the repeats within the F1157CHC genome using Reputer^70,71^.

The genome was analysed with the following Reputer parameters (number of best hits: 10,000; minimum length: 30 bp; and maximum Hamming distance: 3), and the output parsed through a MySQL database with a custom Perl script to generate the tracks to allow the links between all repeated regions to be visualized on the Circos plot. Of the four possible repeats, only the forward and palindromic repeats were detected. Furthermore, depending on the Hamming distance between the two repeats, the links were coloured to show those with a smaller Hamming distance as a darker colour. In order to assess the overall genome architecture in comparison to other *L. longbeachae* genomes, the MAUVE plugin within Geneious (v. 9.1.8) was used to visualize the F1157CHC genome against NSW150 and FDAARGOS_201.

Plasmid architecture was assessed by aligning the five *L. longbeachae* reference plasmids pB1445CHC_73k, pB1445CHC_150k, pB41211CHC_76k, pF1157CHC and pNSW150 and visualising using MAUVE plugin within Geneious (v.9.1.8) as described above.

### *Legionella longbeachae* methylome

Methylated bases were detected for isolate F1157CHC, using the “RS_Modification_and Motif Analysis” protocol implemented in SMRTAnalysis v2.3.0 using the SMRTbell DNA library described above as input. This pipeline takes advantage of BLASR (v1^72^) to map sequencing reads to the assembled genome and MotifFinder v1 to identify sequence motifs associated with particular modifications. The resulting files were submitted to REBASE^73^ along with our annotated reference genome to identify protein coding genes that may be responsible for the inferred methylation patterns.

The distribution of methylated bases on the reference genome, and with regard to genomic features was analysed using bedtools (v2.25.0^74^) and the R statistical language (v3.4). We tested for differences in methylation rate between genes of different functional classes using anova, as implemented in R. A complete record of the code used to perform statistical analyses and visualisation of the methylome data is provided in (S4 file).

## Supplementary Information

Supplementary Information 1.

Supplementary Information 2.

## Data Availability

The raw reads of the 54 New Zealand *L. longbeachae* isolates are available in the NCBI Bioproject database (https://www.ncbi.nlm.nih.gov/bioproject/) under accession number PRJNA417721 and in the Sequence Read Archive (SRA) database (https://www.ncbi.nlm.gov/sra) under accession numbers SRX3379702-SRX3379755. Our complete annotated reference genome for isolate F1157CHC is available in genbank genome (https://www.ncbi.nlm.gov/genome) under accession numbers NZ_CP020894 (chromosome) and NZ_CP020895 (plasmid). All supporting data, code and protocols have been provided within the article or through Supplementary Data.

## References

[1] Phin N (2014). Epidemiology and clinical management of Legionnaires' disease. Lancet Infect. Dis..

[2] Priest PC (2019). The burden of Legionnaires' disease in New Zealand (LegiNZ): A national surveillance study. Lancet Infect. Dis..

[3] Harte, D. *Laboratory-based legionellosis surveillance, 2012*. *New Zealand Public Health Surveillance Report* (2013).

[4] Murdoch DR (2013). Impact of routine systematic polymerase chain reaction testing on case finding for Legionnaires' disease: A pre-post comparison study. Clin. Infect. Dis..

[5] Yu VL (2002). Distribution of *Legionella* species and serogroups isolated by culture in patients with sporadic community-acquired legionellosis: An international collaborative survey. J. Infect. Dis..

[6] Lindsay DS (2012). *Legionella longbeachae* serogroup 1 infections linked to potting compost. J. Med. Microbiol..

[7] Whiley H, Bentham R (2011). *Legionella longbeachae* and legionellosis. Emerg. Infect. Dis..

[8] Kenagy E (2017). Risk factors for *Legionella longbeachae* legionnaires' disease, New Zealand. Emerg. Infect. Dis..

[9] O'Connor BA (2007). Does using potting mix make you sick? Results from a *Legionella longbeachae* case-control study in South Australia. Epidemiol. Infect..

[10] Cazalet C (2010). Analysis of the *Legionella longbeachae* genome and transcriptome uncovers unique strategies to cause Legionnaires' disease. PLoS Genet..

[11] Kozak NA (2010). Virulence factors encoded by *Legionella longbeachae* identified on the basis of the genome sequence analysis of clinical isolate D-4968. J. Bacteriol..

[12] Burstein D (2016). Genomic analysis of 38 *Legionella* species identifies large and diverse effector repertoires. Nat. Genet.

[13] Joseph SJ (2016). Dynamics of genome change among *Legionella* species. Sci. Rep..

[14] Gomez-Valero L (2011). Comparative and functional genomics of legionella identified eukaryotic like proteins as key players in host-pathogen interactions. Front. Microbiol..

[15] den Boer JW (2007). Legionnaires' disease and gardening. Clin. Microbiol. Infect..

[16] Potts A (2013). Cluster of Legionnaires disease cases caused by *Legionella longbeachae* serogroup 1, Scotland, August to September 2013. Euro Surveill..

[17] Bacigalupe R (2017). Population genomics of *Legionella longbeachae* and hidden complexities of infection source attribution. Emerg. Infect. Dis..

[18] McKinney RM (1981). *Legionella longbeachae* species nova, another etiologic agent of human pneumonia. Ann. Intern. Med..

[19] Slow S (2017). Complete genome sequence of a *Legionella longbeachae* serogroup 1 strain isolated from a patient with legionnaires' disease. Genome Announc..

[20] David S (2016). Multiple major disease-associated clones of *Legionella pneumophila* have emerged recently and independently. Genome Res..

[21] Chambers ST (1999). *Legionella,**Chlamydia pneumoniae* and *Mycoplasma* infection in patients admitted to Christchurch Hospital with pneumonia. N. Z. Med. J..

[22] Neill AM (1996). Community acquired pneumonia: aetiology and usefulness of severity criteria on admission. Thorax.

[23] Chambers ST (2020). Pine species provide a niche for *Legionella longbeachae*. J. Appl. Environ. Microbiol..

[24] Hawkins BJ (2010). Relating nutritional and physiological characteristics to growth of *Pinus radiata* clones planted on a range of sites in New Zealand. Tree Physiol..

[25] Huerta-Cepas J (2019). eggNOG 5.0: A hierarchical, functionally and phylogenetically annotated orthology resource based on 5090 organisms and 2502 viruses. Nucleic Acids Res..

[26] Page AJ (2015). Roary: Rapid large-scale prokaryote pan genome analysis. Bioinformatics.

[27] Gomez-Valero L (2019). More than 18,000 effectors in the *Legionella* genus genome provide multiple, independent combinations for replication in human cells. Proc. Natl. Acad. Sci. USA.

[28] Timms VJ (2018). Genome sequencing links persistent outbreak of Legionellosis in Sydney (New South Wales, Australia) to an emerging clone of *Legionella pneumophila* sequence type 211. Appl. Environ. Microbiol..

[29] Zhang J (2018). Genome-by-genome approach for fast bacterial genealogical relationship evaluation. Bioinformatics.

[30] Letunic I, Bork P (2019). Interactive tree of life (iTOL) v4: Recent updates and new developments. Nucleic Acids Res..

[31] Franceschini N (2001). Characterization of OXA-29 from *Legionella (Fluoribacter) gormanii*: Molecular class D beta-lactamase with unusual properties. Antimicrob. Agents Chemother..

[32] Avison MB, Simm AM (2002). Sequence and genome context analysis of a new molecular class D beta-lactamase gene from *Legionella pneumophila*. J. Antimicrob. Chemother..

[33] Forsberg KJ (2015). The tetracycline destructases: A novel family of tetracycline-inactivating enzymes. Chem. Biol..

[34] Markley JL, Wencewicz TA (2018). Tetracycline-inactivating enzymes. Front. Microbiol..

[35] Isenman H (2018). Antimicrobial susceptibilities of clinical *Legionella longbeachae* isolates. J. Antimicrob. Chemother..

[36] Qin T (2017). Distribution of secretion systems in the genus *Legionella* and its correlation with pathogenicity. Front. Microbiol..

[37] Goessweiner-Mohr N (2014). Conjugation in gram-positive bacteria. Microbiol. Spectr..

[38] Brunel R, Charpentier X (2016). Trans-translation is essential in the human pathogen *Legionella pneumophila*. Sci. Rep..

[39] El-Gebali S (2019). The Pfam protein families database in 2019. Nucleic Acids Res..

[40] Williams KP (2002). Integration sites for genetic elements in prokaryotic tRNA and tmRNA genes: Sublocation preference of integrase subfamilies. Nucleic Acids Res..

[41] Slow S (2018). Complete genome sequence of *Legionella sainthelensi* isolated from a patient with legionnaires' disease. Genome Announc..

[42] Koonin EV, Makarova KS (2017). Mobile genetic elements and evolution of CRISPR-cas systems: All the way there and back. Genome Biol. Evol..

[43] Makarova KS (2015). An updated evolutionary classification of CRISPR-Cas systems. Nat. Rev. Microbiol..

[44] Pinilla-Redondo R (2020). Type IV CRISPR-Cas systems are highly diverse and involved in competition between plasmids. Nucleic Acids Res..

[45] Deecker SR, Ensminger AW (2020). Type I-F CRISPR-cas distribution and array dynamics in *Legionella pneumophila*. G3.

[46] Zhang Y (2017). Benefits of genomic insights and CRISPR-cas signatures to monitor potential pathogens across drinking water production and distribution systems. Front. Microbiol..

[47] Beaulaurier J (2018). Metagenomic binning and association of plasmids with bacterial host genomes using DNA methylation. Nat. Biotechnol..

[48] Langmead B, Salzberg SL (2012). Fast gapped-read alignment with Bowtie 2. Nat. Methods.

[49] Cox MP, Peterson DA, Biggs PJ (2010). SolexaQA: At-a-glance quality assessment of Illumina second-generation sequencing data. BMC Bioinform..

[50] Bankevich A (2012). SPAdes: A new genome assembly algorithm and its applications to single-cell sequencing. J. Comput. Biol..

[51] Li H, Durbin R (2009). Fast and accurate short read alignment with Burrows–Wheeler transform. Bioinformatics.

[52] Li H (2011). A statistical framework for SNP calling, mutation discovery, association mapping and population genetical parameter estimation from sequencing data. Bioinformatics.

[53] Garrison E. M. G. *Haplotype-Based Variant Detection from Short-Read Sequencing.* http://arxiv.org/abs/1207.3097. (2012).

[54] Croucher NJ (2015). Rapid phylogenetic analysis of large samples of recombinant bacterial whole genome sequences using Gubbins. Nucleic Acids Res..

[55] Bouckaert R (2014). BEAST 2: A software platform for Bayesian evolutionary analysis. PLoS Comput. Biol..

[56] Bouckaert RR, Drummond AJ (2017). bModelTest: Bayesian phylogenetic site model averaging and model comparison. BMC Evol. Biol..

[57] Russel PM (2019). Model selection and parameter inference in phylogenetics using nested sampling. Syst. Biol..

[58] Baele G (2012). Improving the accuracy of demographic and molecular clock model comparison while accommodating phylogenetic uncertainty. Mol. Biol. Evol..

[59] American Mathematical Society. Some probabilistic and statistical problems in the analysis of DNA sequences. *Some mathematical questions in Biology: DNA sequence analysis*, vol. 17. (American Mathematical Society, 1986).

[60] Drummond AJ (2006). Relaxed phylogenetics and dating with confidence. PLoS Biol..

[61] Biek R (2015). Measurably evolving pathogens in the genomic era. Trends Ecol. Evol..

[62] He Z (2016). Evolview v2: An online visualization and management tool for customized and annotated phylogenetic trees. Nucleic Acids Res..

[63] Stamatakis A (2014). RAxML version 8: A tool for phylogenetic analysis and post-analysis of large phylogenies. Bioinformatics.

[64] Huerta-Cepas J (2017). Fast genome-wide functional annotation through orthology assignment by eggNOG-Mapper. Mol. Biol. Evol..

[65] Seemann T (2014). Prokka: Rapid prokaryotic genome annotation. Bioinformatics.

[66] Huson DH (1998). SplitsTree: Analyzing and visualizing evolutionary data. Bioinformatics.

[67] Kloepper TH, Huson DH (2008). Drawing explicit phylogenetic networks and their integration into SplitsTree. BMC Evol. Biol..

[68] Sullivan MJ, Petty NK, Beatson SA (2011). Easyfig: A genome comparison visualizer. Bioinformatics.

[69] Krzywinski M (2009). Circos: An information aesthetic for comparative genomics. Genome Res..

[70] Kurtz S (2001). REPuter: The manifold applications of repeat analysis on a genomic scale. Nucleic Acids Res..

[71] Kurtz S, Schleiermacher C (1999). REPuter: Fast computation of maximal repeats in complete genomes. Bioinformatics.

[72] Chaisson MJ, Tesler G (2012). Mapping single molecule sequencing reads using basic local alignment with successive refinement (BLASR): Application and theory. BMC Bioinform..

[73] Lim Y-L (2016). Complete genome sequence and methylome analysis of *Aeromonas hydrophila* strain YL17 isolated from a compost pile. Genome Announc..

[74] Quinlan AR, Hall IM (2010). BEDTools: A flexible suite of utilities for comparing genomic features. Bioinformatics.

