# Supplementary material for: Extensive epigenetic modification with large-scale chromosomal and plasmid recombination characterise the Legionella longbeachae serogroup 1 genome
Source: Sci Rep. 2022 Apr 6;12:5810. doi: 10.1038/s41598-022-09721-9 (PMC8987031; doi:10.1038/s41598-022-09721-9)
Supplement: Supplementary file 1 — Supplementary Information 1. [file 41598_2022_9721_MOESM1_ESM.pdf]

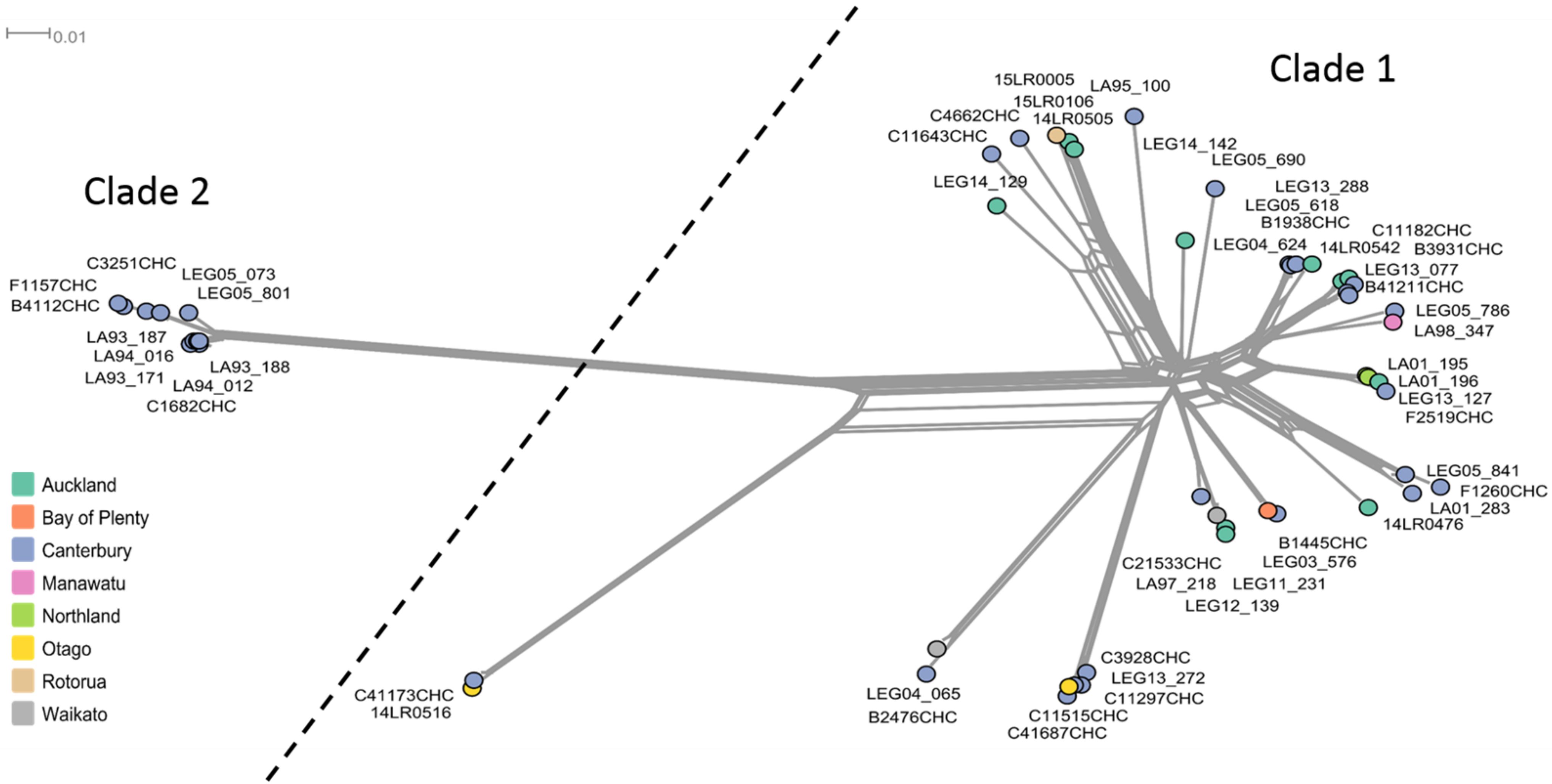

Supplementary Figure 1: NeighbourNet tree of 54 *L. longbeachae* clinical isolates based on 1,271 core SNPs. The scale bar represents the number of nucleotide substitutions/site and the isolates are coloured by region as depicted in the key.

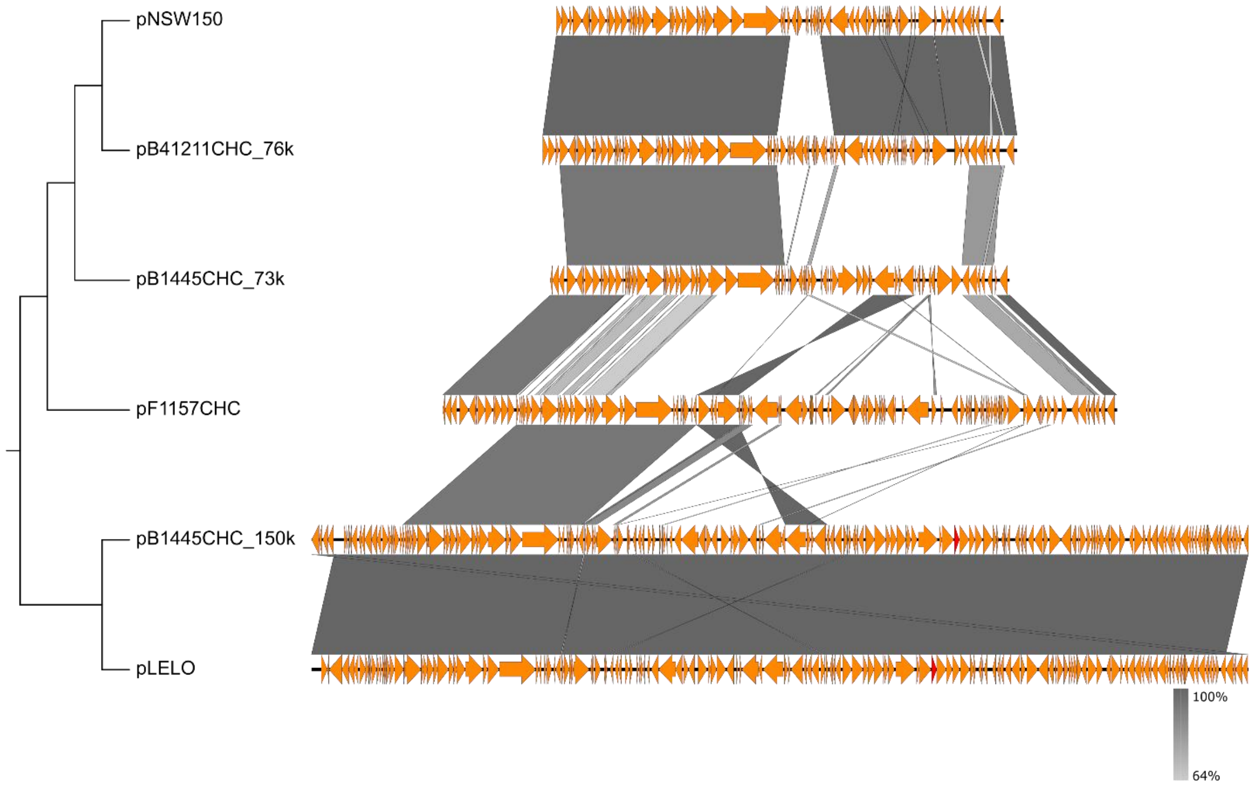

Supplementary Figure 2: Dendrogram of *L. longbeachae* reference plasmid sequences based on gene presence-absence, and alignment of sequences.

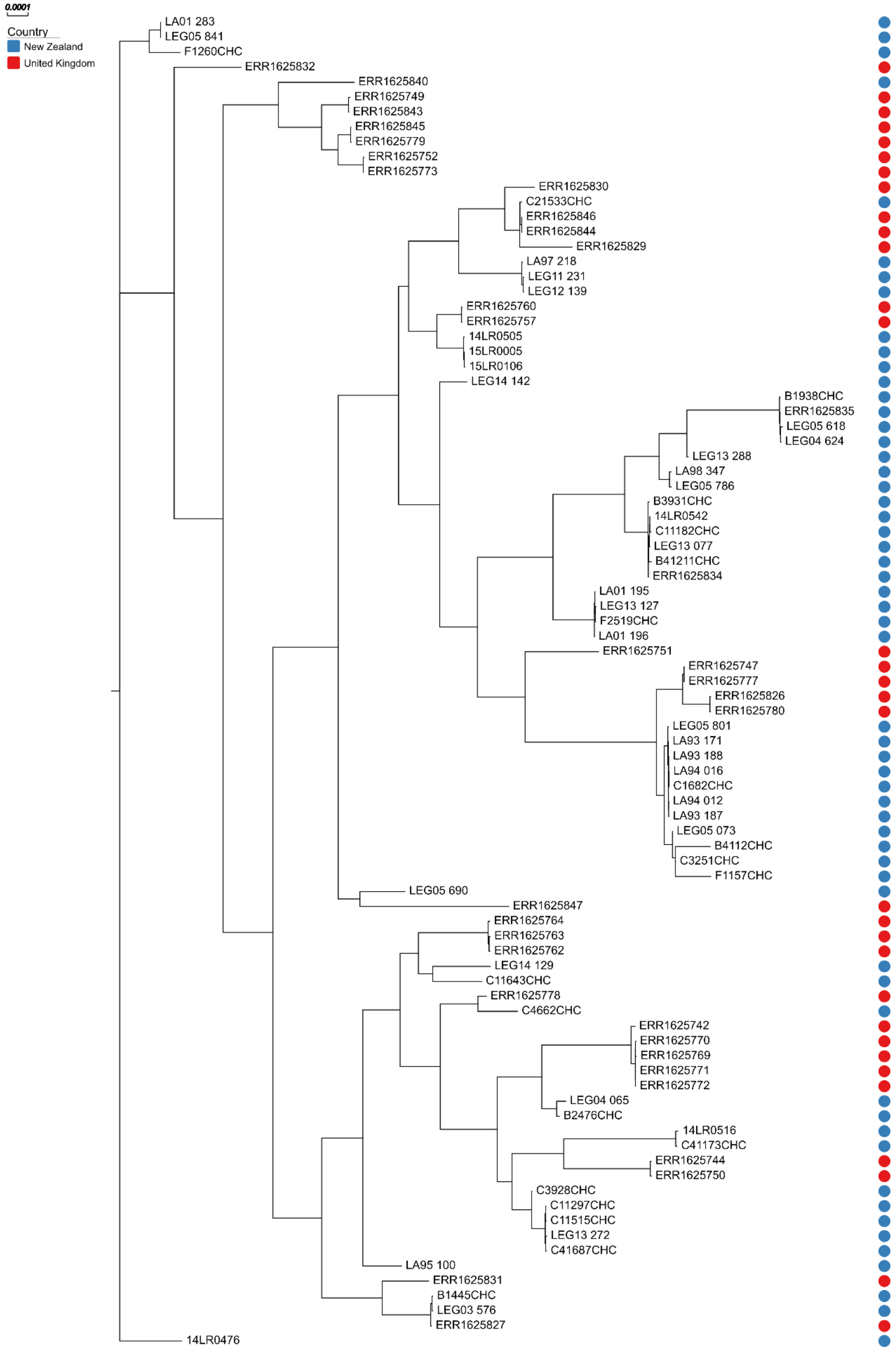

Supplementary Figure 3: Maximum likelihood tree of 89 *L. longbeachae* isolates from New Zealand and the United Kingdom. The scale bar represents the proportion of substitutions. The isolates are coloured by country.

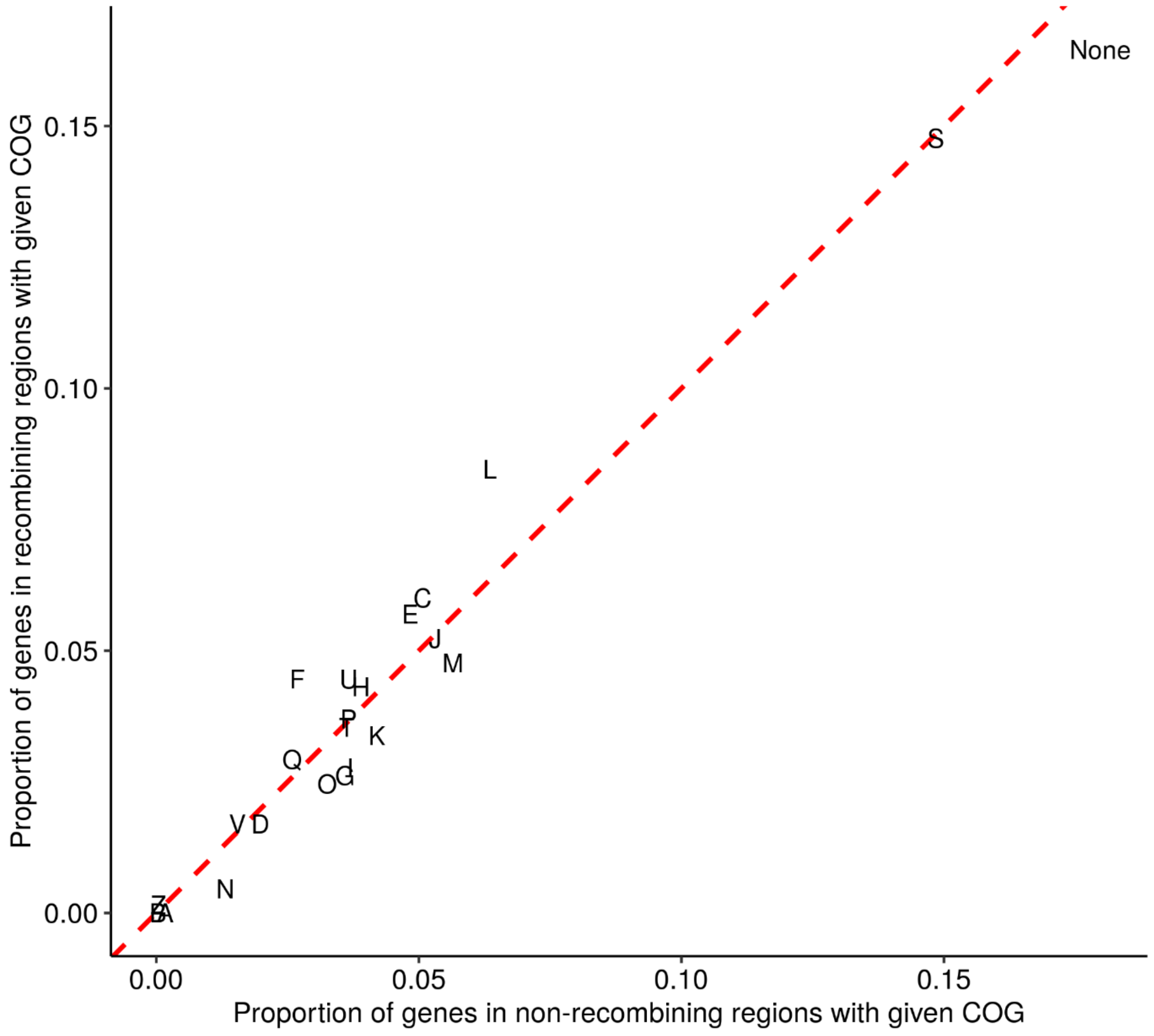

S4 Figure: Regions of recombination and non-recombination by COG category**.** Each letter represents the proportion of genes falling in high recombination regions (y-axis) and non-recombining regions (x-axis) annotated to a specific COG category. The dashed red line represents the expected values if no differences exist between the proportion of genes in regions of recombination and non-recombination with given COG. COG categories are represented using their one-letter abbreviation. J, translation, L, replication, recombination and repair; K, transcription; O, molecular chaperones and related functions; M, cell wall structure and biogenesis and outer membrane; N, secretion, motility and chemotaxis; T, signal transduction; P, inorganic ion transport and metabolism; C, energy production and conversion; G, carbohydrate metabolism and transport; E, amino acid metabolism and transport; F, nucleotide metabolism and transport; H, coenzyme metabolism; I, lipid metabolism; D, cell division and chromosome partitioning; R, general functional prediction only; S, no functional prediction.

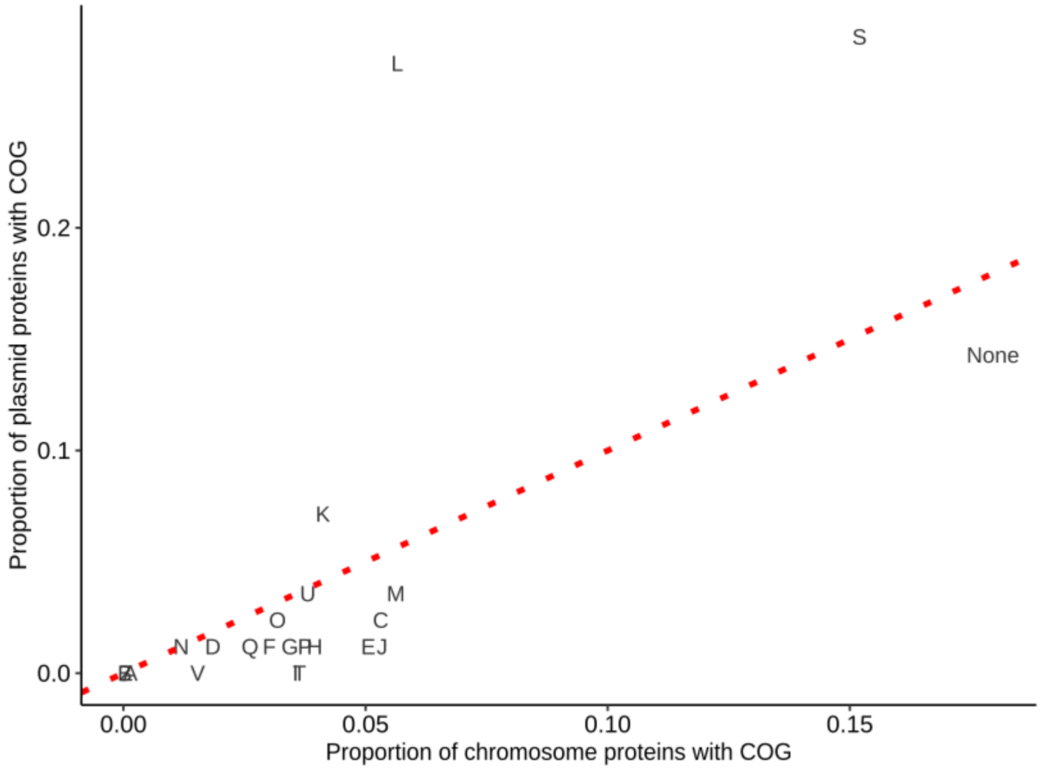

S5 Figure: Proportion of chromosome and plasmid encoded genes by COG category. Each letter represents the proportion of chromosome-encoded (x-axis) and plasmid-encoded (y-axis) genes annotated to a specific COG category. The dashed red line represents the expected values if no differences exist between chromosome and plasmid. COG categories are represented using their one-letter abbreviation. J, translation, L, replication, recombination and repair; K, transcription; O, molecular chaperones and related functions; M, cell wall structure and biogenesis and outer membrane; N, secretion, motility and chemotaxis; T, signal transduction; P, inorganic ion transport and metabolism; C, energy production and conversion; G, carbohydrate metabolism and transport; E, amino acid metabolism and transport; F, nucleotide metabolism and transport; H, coenzyme metabolism; I, lipid metabolism; D, cell division and chromosome partitioning; R, general functional prediction only; S, no functional prediction.

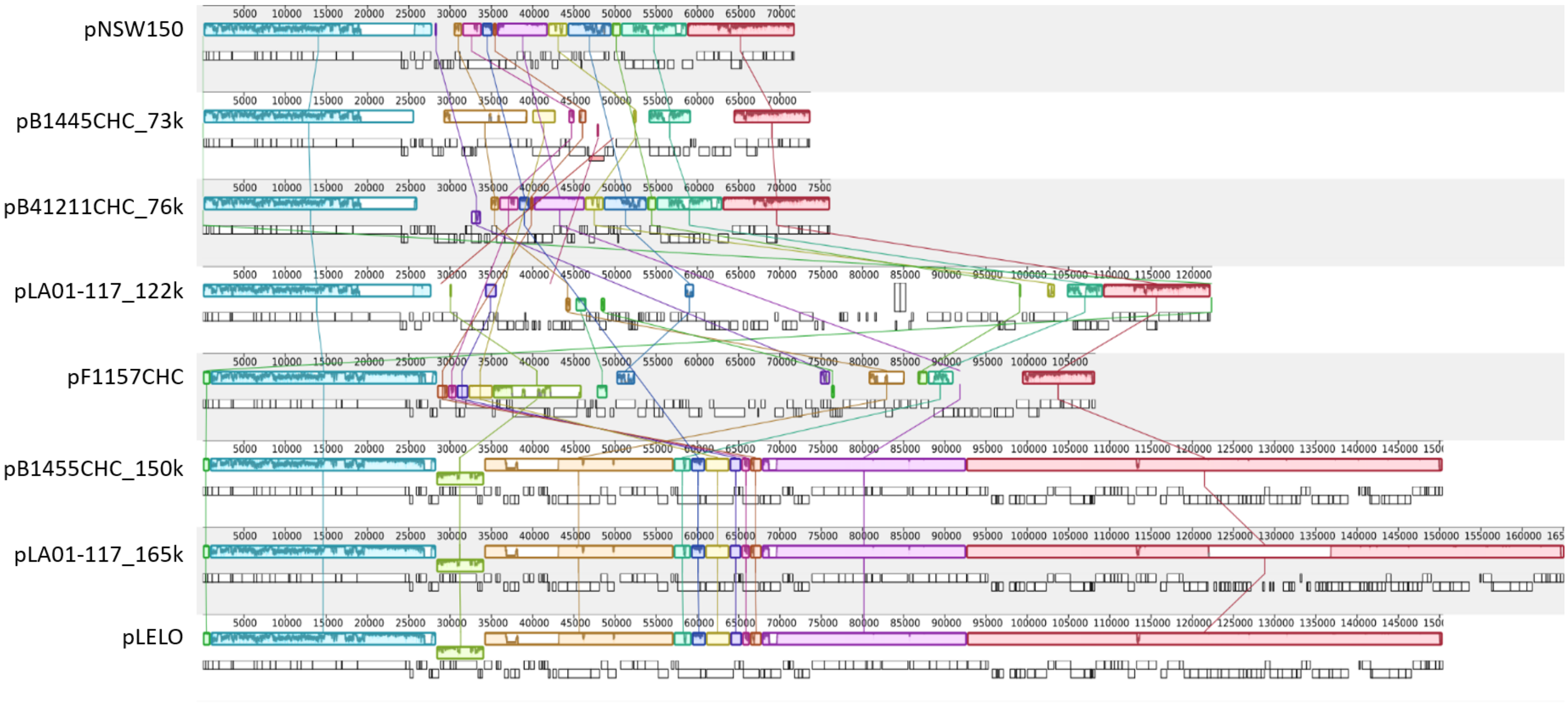

Supplementary Figure 6: Mauve alignment of *Legionella* plasmids. Plasmids can be broadly divided into two groups. The first group consisting of plasmids ~ 70 kb in size were found only in *L. longbeachae* isolates, while the second group of plasmids ~ 150 kb in size were found in isolates from several different *Legionella* species.

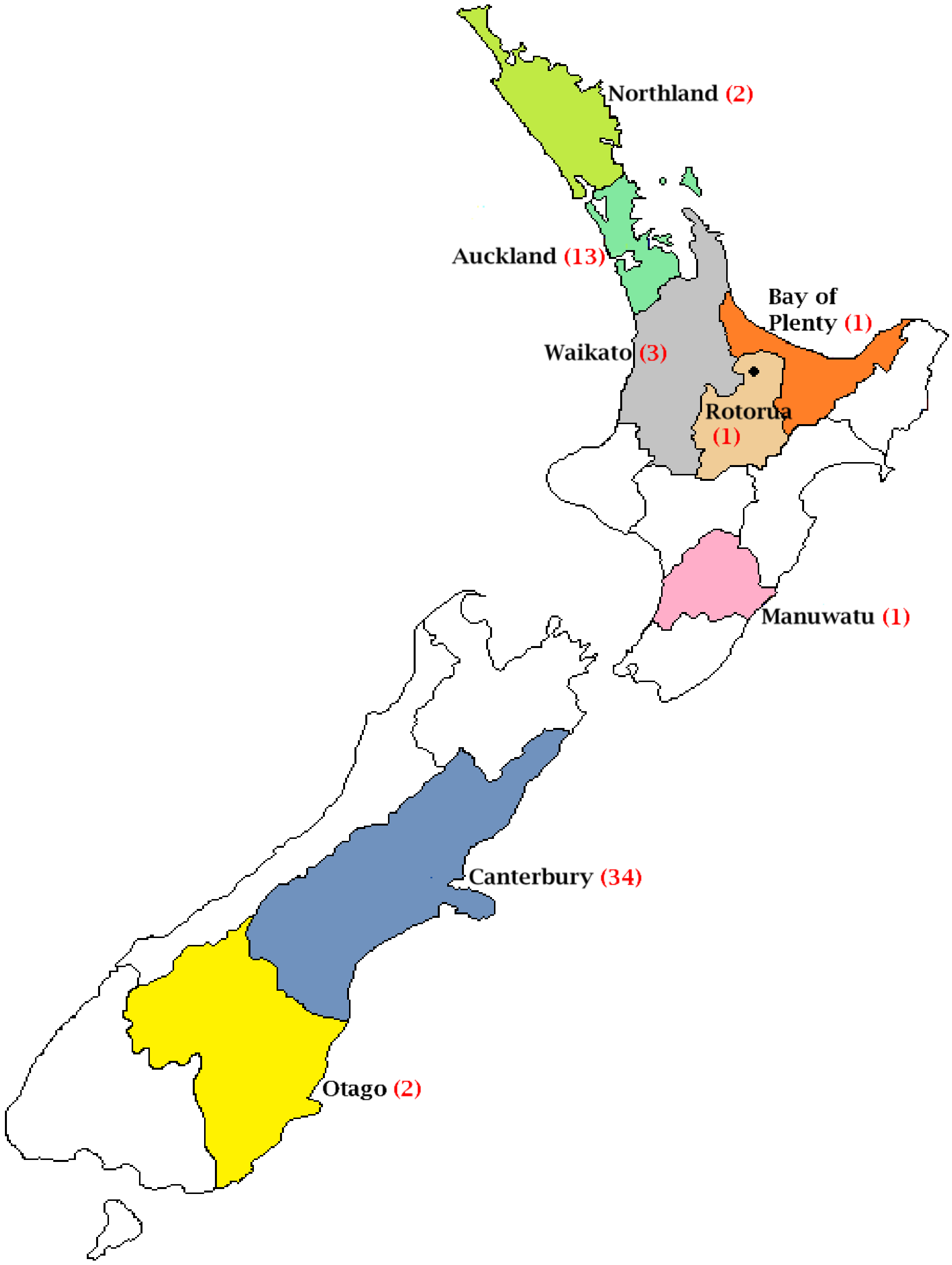

Supplementary Figure 7: Regional Origin of the Sequenced Isolates. The number of isolates sequenced from each region is shown in brackets. NZ map and DHB boundaries obtained from:
https://services.arcgis.com/X6pbMkffRhVOzZha/arcgis/rest/services/DHB_NZ/FeatureServer

Supplementary Table 2**:** Meta- and genome summary data from Prokka of the *Legionella longbeachae* clinical isolates sequenced and analysed in the current study.

Sample ID	Sample Type	DFA	Date Isolated	Region	Source	Reads	Length	Contigs	%GC	Sig Peptide	CDS	rRNA	tRNA	tmRNA
LA93/171	TA	sg1	3/11/1993	Canterbury	ESR	1,255,382	4,151,384	124	37.09	273	3,585	7	45	1
LA93/187	TA	sg1	23/11/1993	Canterbury	ESR	1,287,057	4,153,978	121	37.09	274	3,587	7	45	1
LA93/188	Sputum	sg1	24/11/1993	Canterbury	ESR	1,639,517	4,154,783	127	37.09	275	3,585	7	45	1
LA94/012	Sputum	sg1	26/01/1994	Canterbury	ESR	1,215,358	4,156,823	134	37.09	273	3,591	7	45	1
LA94/016	Sputum	sg1	1/02/1994	Canterbury	ESR	1,098,891	4,146,730	110	37.08	274	3,578	7	45	1
LA95/100	Sputum	sg1	14/11/1995	Canterbury	ESR	1,512,101	3,955,145	67	37.06	259	3,385	7	44	1
LA97/218	Culture	sg1	29/10/1997	Waikato	ESR	1,558,944	4,135,249	87	37.09	280	3,593	7	44	1
LA98/347	Culture	sg1	29/10/1998	Manawatu	ESR	1,964,078	4,086,232	85	37.10	274	3,526	7	44	1
LA01/195	Culture	sg1	31/07/2001	Northland	ESR	1,218,634	4,215,808	70	37.11	279	3,638	7	44	1
LA01/196	Culture	sg1	31/07/2001	Northland	ESR	936,419	4,213,829	78	37.12	279	3,637	7	44	1
LA01/283	Culture	sg1	7/11/2001	Canterbury	ESR	1,227,702	4,116,538	53	37.11	274	3,550	7	44	1
LEG03/576	TA	sg1	11/09/2003	Bay of Plenty	ESR	1,037,475	4,204,256	77	37.17	285	3,639	6	44	1
LEG04/065	Culture	sg1	13/02/2004	Waikato	ESR	1,270,187	4,186,661	106	37.16	289	3,640	7	44	1
LEG04/624	Culture	sg1	21/10/2004	Canterbury	ESR	1,444,358	4,022,107	65	37.11	270	3,446	7	44	1
LEG05/073	Sputum	sg1	10/02/2005	Canterbury	ESR	1,488,662	4,138,776	110	37.08	276	3,567	7	44	1
LEG05/618	Culture	sg1	4/10/2005	Canterbury	ESR	1,666,151	4,036,735	103	37.12	271	3,456	7	41	1
LEG05/690	Culture	sg1	26/10/2005	Canterbury	ESR	1,637,748	4,104,723	92	37.10	275	3,547	7	44	1
LEG05/786	Culture	sg1	25/11/2005	Canterbury	ESR	1,603,650	4,102,992	109	37.13	276	3,514	7	44	1
LEG05/801	Culture	sg1	1/12/2005	Canterbury	ESR	884,331	4,155,276	150	37.09	274	3,592	7	45	1
LEG05/841	Culture	sg1	12/12/2005	Canterbury	ESR	694,402	4,118,421	69	37.12	273	3,555	7	44	1
LEG11/231	Culture	sg1	11/05/2011	Auckland	ESR	726,684	4,222,808	85	37.12	291	3,664	7	44	1
LEG12/139	TA	sg1	10/02/2012	Auckland	ESR	1,020,081	4,256,291	212	37.22	291	3,702	7	49	1
LEG13/077	Culture	sg1	24/01/2013	Auckland	ESR	1,473,821	4,159,265	416	37.35	278	3,563	6	50	1
LEG13/127	Sputum	sg1	7/03/2013	Auckland	ESR	1,446,270	4,258,466	68	37.12	273	3,677	7	44	1
LEG13/272	Culture	sg1	30/01/2013	Otago	ESR	1,034,580	4,209,908	305	37.24	284	3,626	7	50	1
LEG13/288	Culture	sg1	16/08/2013	Auckland	ESR	1,006,254	4,076,887	93	37.12	284	3,498	7	44	1
LEG14/129	Culture	sg1	18/02/2014	Auckland	ESR	1,099,344	4,162,158	74	37.15	281	3,595	7	44	1
LEG14/142	Culture	sg1	26/02/2014	Auckland	ESR	1,142,960	4,106,547	77	37.07	270	3,517	7	45	1

14LR0476	TA	sg1	30/09/2014	Auckland	ESR	1,200,522	4,188,904	66	37.13	280	3,691	7	44	1
14LR0505	RULL	sg1	29/10/2014	Auckland	ESR	977,565	4,049,226	85	37.1	278	3,463	7	44	1
14LR0516	Sputum	sg1	7/11/2014	Otago	ESR	820,679	4,186,788	720	37.35	261	3,713	7	46	1
14LR0542	Sputum	sg1	20/11/2014	Auckland	ESR	925,853	3,978,976	68	37.09	278	3,406	6	44	1
15LR0005	Sputum	sg1	7/01/2015	Rotorua	ESR	1,618,978	4,173,022	468	37.4	276	3,564	8	50	1
15LR0106	Sputum	sg1	5/03/2015	Auckland	ESR	1,605,409	4,202,962	663	37.50	272	3,585	7	58	1
B1445CHC	Sputum	sg2	18/01/2007	Canterbury	CHL	1,481,893	4,341,256	610	37.51	284	3,759	5	57	1
B1938CHC	Sputum	sg1	15/05/2007	Canterbury	CHL	621,278	4,030,052	75	37.11	272	3,484	7	44	1
B2476CHC	Sputum	sg1	19/01/2008	Canterbury	CHL	948,241	4,272,377	129	37.17	297	3,737	7	45	1
B4112CHC	TA	sg1	19/12/2009	Canterbury	CHL	987,258	4,220,732	122	37.09	279	3,650	7	43	1
B3931CHC	Sputum	sg1	22/01/2009	Canterbury	CHL	435,509	4,094,453	79	37.12	278	3,526	6	44	1
B41211CHC	Sputum	sg1	11/01/2010	Canterbury	CHL	1,168,782	4,049,178	87	37.10	272	3,479	7	43	1
C1682CHC	TA	sg1	29/07/2010	Canterbury	CHL	1,118,390	4,156,161	137	37.09	273	3,593	7	45	1
C11182CHC	Sputum	sg1	10/11/2010	Canterbury	CHL	970,420	4,050,292	83	37.11	274	3,474	6	44	1
C11297CHC	Sputum	sg1	25/11/2010	Canterbury	CHL	1,534,564	4,204,266	75	37.12	291	3,627	7	44	1
C11515CHC	Sputum	sg1	28/12/2010	Canterbury	CHL	1,062,618	4,209,631	80	37.14	293	3,633	7	44	1
C11643CHC	Sputum	sg1	18/01/2011	Canterbury	CHL	851,898	4,174,650	66	37.15	273	3,604	7	44	1
C3251CHC	Sputum	sg1	26/10/2011	Canterbury	CHL	868,513	4,186,229	114	37.08	280	3,613	7	44	1
C21533CHC	Sputum	sg1	09/11/2011	Canterbury	CHL	1,145,828	4,076,539	73	37.07	276	3,503	7	44	1
C3928CHC	Sputum	sg1	01/02/2012	Canterbury	CHL	1,004,185	4,186,320	95	37.11	287	3,592	7	44	1
C4662CHC	Sputum	sg1	04/09/2012	Canterbury	CHL	2,031,908	4,001,848	67	37.09	269	3,431	7	44	1
C41173CHC	TA	sg1	25/11/2012	Canterbury	CHL	1,065,823	4,218,769	127	37.16	279	3,628	7	44	1
C41687CHC	Sputum	sg1	29/01/2013	Canterbury	CHL	1,206,072	4,163,199	80	37.13	287	3,580	7	44	1
F1260CHC	Sputum	sg1	28/10/2013	Canterbury	CHL	1,171,339	4,203,778	62	37.16	282	3,635	7	45	1
F1157CHC[*]	Sputum	sg1	28/04/2014	Canterbury	CHL	1,157,694	4,271,029	2	37.15	289	3,695	12	52	1
F2519CHC	Sputum	sg1	04/07/2014	Canterbury	CHL	890,835	4,215,940	66	37.11	280	3,641	7	44	1
NSW150							4,149158	2	37.13	279	3,546	12	46	1
FDAARGOS_201							4,162,717	1	37.06	280	3,588	12	47	1
LA99_361[‡]	Culture	sg1	1/12/1999	Waikato	ESR	74,325	4,238,528	381	37.16		4,956			
LA01_117[‡**]	Culture	sg1	9/04/2001	Canterbury	ESR	1,626,572	4,560,575	96	36.81		4,015			
LEG13_514[‡]	Sputum	sg2	20/12/2013	Bay of Plenty	ESR	776,842	4,199,513	69	37.14		3,741			

B3526CHC‡	LLLA	sg1or2	14/11/08	Canterbury	CHL	893,279	4,169,413	68	37.10	3,703
LEG11_751‡	Culture	sg1	21/11/2011	Auckland	ESR	1,052,266	4,334,205	694	37.61	5,916
LEG12_138‡	Sputum	sg1	9/02/2012	Auckland	ESR	1,482,579	4,442,725	883	37.7	6,596

DFA = Direct Fluorescent Antibody assay result.

TA = Tracheal Aspirate.

RULL = Right Upper Lung Lobe

LLLA = Left Lower Lobe Aspirate

Source = collection where isolates where obtained: ESR- National *Legionella* Reference Laboratory, Institute Environmental Scientific Research, Porirua, New Zealand; CHL-Canterbury Health Laboratories, Christchurch, New Zealand.

Reads = number of sequenced reads obtained from the Illumina MiSeq

Length = the combined length of the contigs used for genome assembly

Contig = the number of contigs used for genome assembly

% GC = the GC content of the contigs used for genome assembly

CDS = the number of coding sequences predicted in the genome

*Isolate was further sequenced using the PacBio RSII system to generate our own complete NZ reference genome (Slow *et al*., 2017).

‡Isolates sequenced but not used in the analysis (Leg13_514 and B3526CHC were sg2 isolates).

**Isolate had been mis-identified and was found to be *Legionella sainthelensi* (Slow *et al*., 2018).

Supplementary Table 3: MIC_{90} (mg/L) values for *L. longbeachae* isolates by broth dilution (BYE; Isenman et al., 2018)

Sample ID	Azithromycin	Clarithromycin	Ciprofloxacin	Moxifloxacin	Rifampicin	Tetracycline
B4112CHC	0.0625	0.03	0.03	0.008	>0.002	16
B1445CHC	0.0625	0.0625	0.03	0.03	0.004	16
B3931CHC	0.03	0.03	0.016	0.008	0.004	32
B41211CHC	0.0625	0.03	0.016	0.016	0.016	16
B2476CHC	0.125	0.0625	0.03	0.03	0.008	32
C3251CHC	0.0625	0.125	0.03	0.03	0.008	16
C41173CHC	0.0625	0.0625	0.03	0.016	0.004	32
C11515CHC	0.0625	0.0625	0.03	0.03	0.002	32
C3928CHC	0.125	0.016	0.016	0.008	0.002	32
C11643CHC	0.0625	0.03	0.008	0.016	0.002	64
C11182CHC	0.0625	0.125	0.016	0.016	0.03	32
C4662CHC	0.0625	0.0625	0.03	0.016	0.016	32
C11297CHC	0.0625	0.0625	0.03	0.016	0.016	16
F1260CHC	0.03	0.0625	0.016	0.016	0.016	64
F1157CHC	0.0625	0.0625	0.03	0.016	0.03	64
F2519CHC	0.0625	0.008	>0.008	>0.008	0.008	32

Supplementary Table 6**:** Sg1 clinical and environmental isolates from Bacigalupae *et al.*, 2017[†] that were included in the global phylogenetic analysis

Sample ID	Accession	Run ID	Date*	Source	Country
04.2845	ERX1696107	ERR1625742	7/6/2004	Patient	United Kingdom
08.1921	ERX1696109	ERR1625744	1/4/2008	Patient	United Kingdom
09.5279	ERX1696112	ERR1625747	20/5/2009	Patient	United Kingdom
09.6863	ERX1696114	ERR1625749	19/11/2009	Compost	United Kingdom
10.4571	ERX1696115	ERR1625750	19/3/2010	Patient	United Kingdom
11.3483(3)	ERX1696116	ERR1625751	13/5/2011	Compost	United Kingdom
11.3484(1)	ERX1696117	ERR1625752	13/5/2011	Compost	United Kingdom
13.8641	ERX1696192	ERR1625827	18/6/2012	Compost	United Kingdom
13.8643	ERX1696194	ERR1625829	18/6/2012	Compost	United Kingdom
13.8644	ERX1696195	ERR1625830	18/6/2012	Compost	United Kingdom
13.8645	ERX1696196	ERR1625831	18/6/2012	Compost	United Kingdom
13.8646	ERX1696197	ERR1625832	26/6/2013	Compost	United Kingdom
13.59701	ERX1696122	ERR1625757	23/8/2013	Patient	United Kingdom
13.59704	ERX1696125	ERR1625760	27/8/2013	Patient	United Kingdom
13.6121	ERX1696127	ERR1625762	30/8/2013	Patient	United Kingdom
13.61211	ERX1696128	ERR1625763	30/8/2013	Patient	United Kingdom
13.61212	ERX1696129	ERR1625764	30/8/2013	Patient	United Kingdom
13.64721	ERX1696134	ERR1625769	13/9/2013	Patient	United Kingdom
13.64722	ERX1696135	ERR1625770	13/9/2013	Patient	United Kingdom
13.64723	ERX1696136	ERR1625771	17/9/2013	Patient	United Kingdom
13.64724	ERX1696137	ERR1625772	19/9/2013	Patient	United Kingdom
13.6614	ERX1696191	ERR1625826	19/9/2013	Compost	United Kingdom
13.6619	ERX1696138	ERR1625773	20/9/2013	Compost	United Kingdom
13.6762	ERX1696142	ERR1625777	25/9/2013	Compost	United Kingdom
13.6763	ERX1696143	ERR1625778	1/10/2013	Compost	United Kingdom
13.6764	ERX1696144	ERR1625779	1/10/2013	Compost	United Kingdom
13.6912	ERX1696145	ERR1625780	29/5/2014	Compost	United Kingdom
8702918	ERX1696208	ERR1625843	8/5/2014	Patient	United Kingdom
8702860	ERX1696209	ERR1625844	9/5/2014	Soil	United Kingdom
8702861	ERX1696210	ERR1625845	10/5/2014	Soil	United Kingdom
8702862	ERX1696211	ERR1625846	1/4/2008	Soil	United Kingdom
8702863	ERX1696212	ERR1625847	17/4/2008	Soil	United Kingdom
13.8293	ERX1696199	ERR1625834	2010	Patient	New Zealand
13.8294	ERX1696200	ERR1625835	2004	Patient	New Zealand
13.8299	ERX1696205	ERR1625840	2003	Compost	New Zealand

[†]Bioproject: PRJEB14754; SRA study: ERP016422

*Date received in the reference laboratory

S1 File: R-plots-Roary outputs file

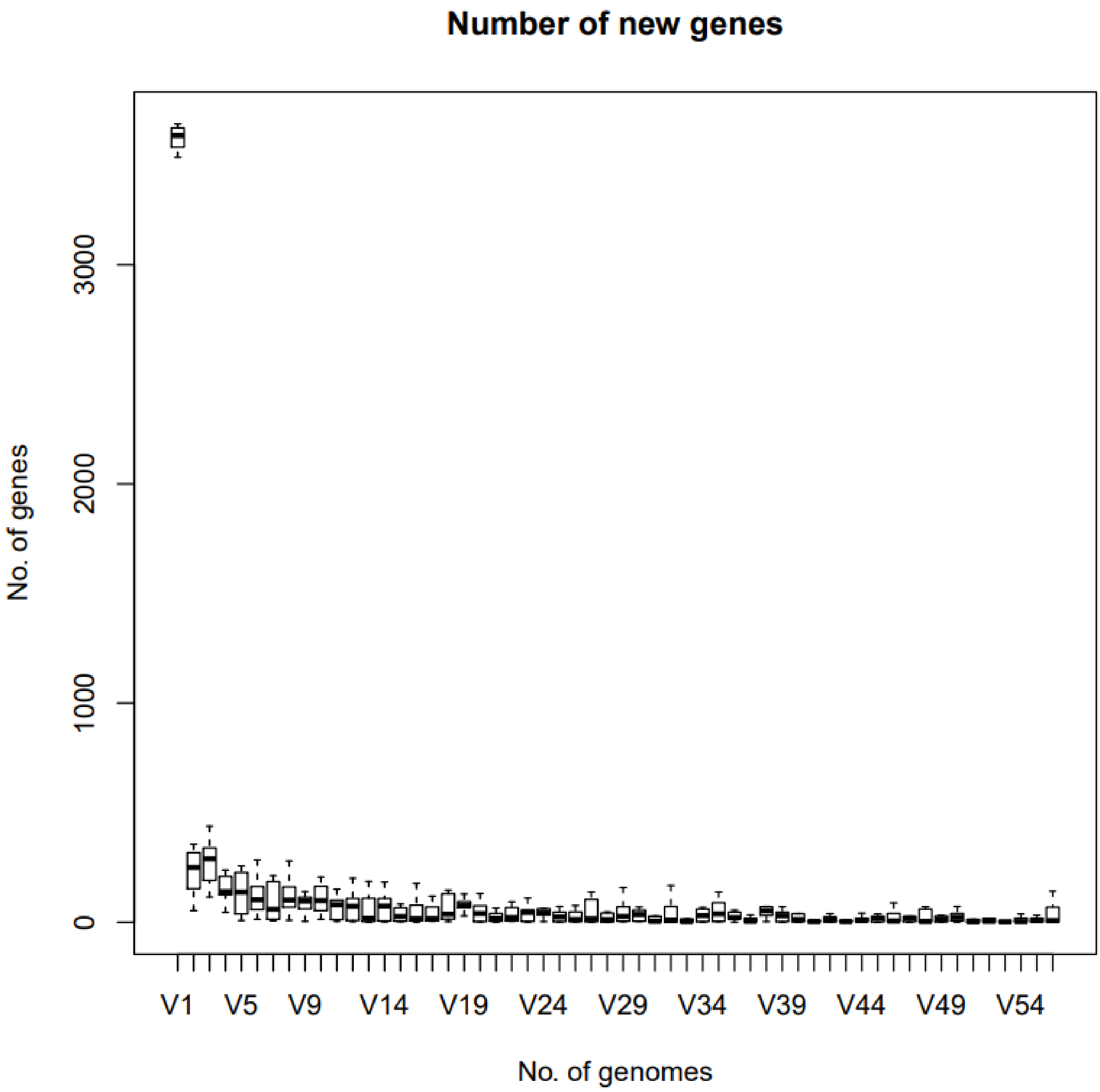

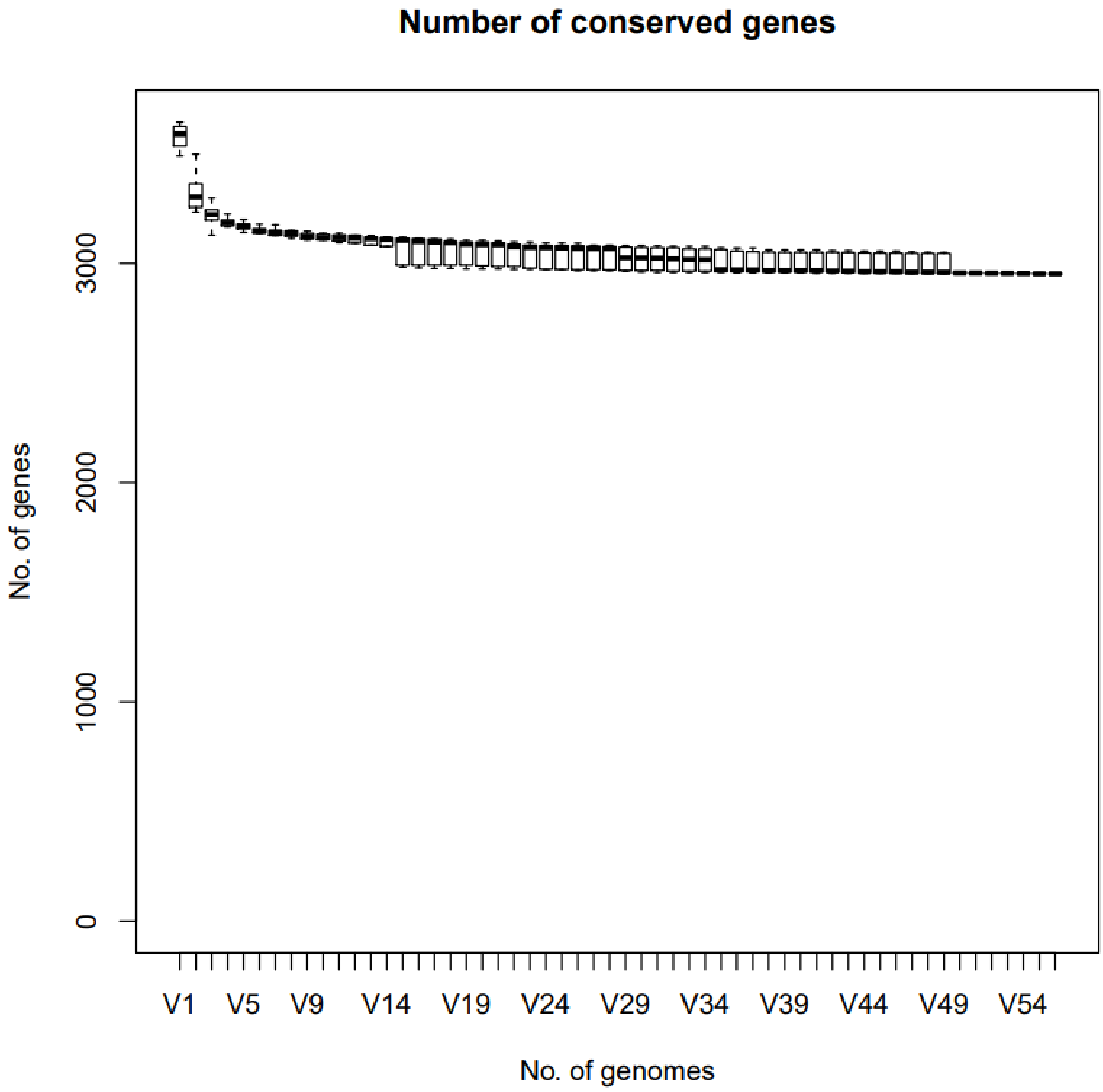

Number of conserved genes
No. of genes
0
1000
2000
3000
V1
V5
V9
V14
V19
V24
V29
V34
V39
V44
V49
V54
No. of genomes

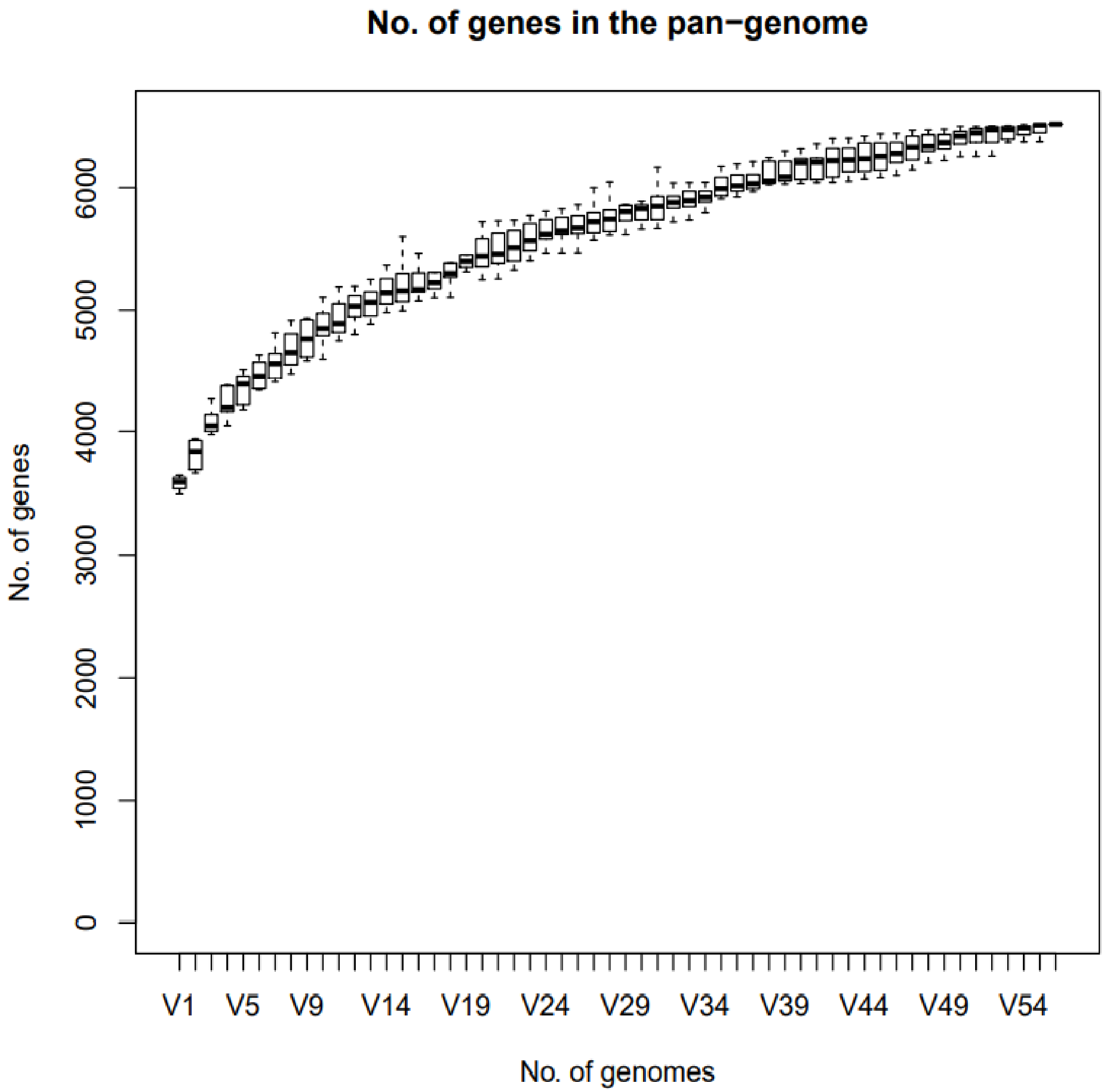
No. of genes in the pan−genome
No. of genes
0
1000
2000
3000
4000
5000
6000
V1
V5
V9
V14
V19
V24
V29
V34
V39
V44
V49
V54
No. of genomes

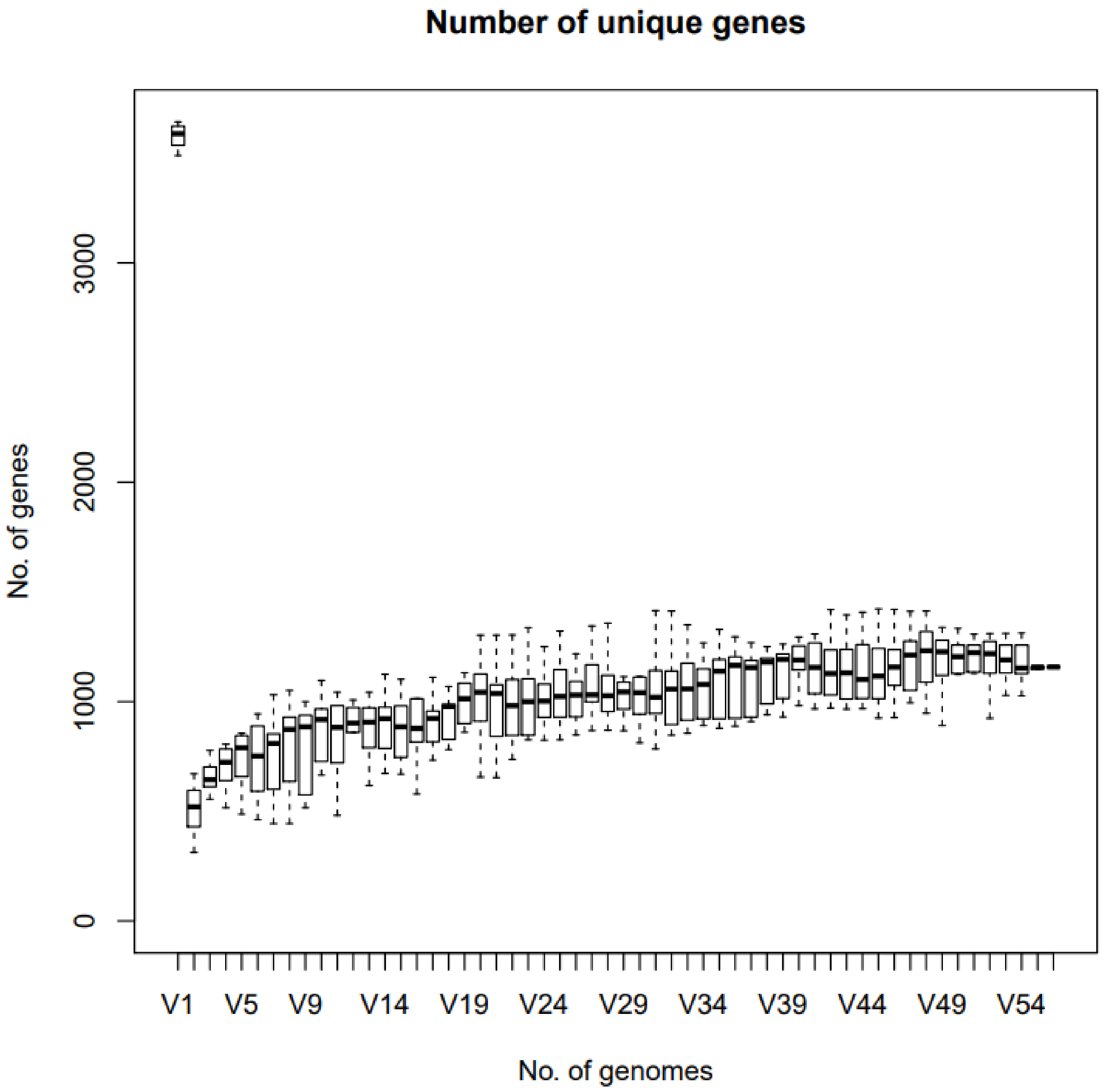
Number of unique genes
No. of genes
0
1000
2000
3000
V1
V5
V9
V14
V19
V24
V29
V34
V39
V44
V49
V54
No. of genomes

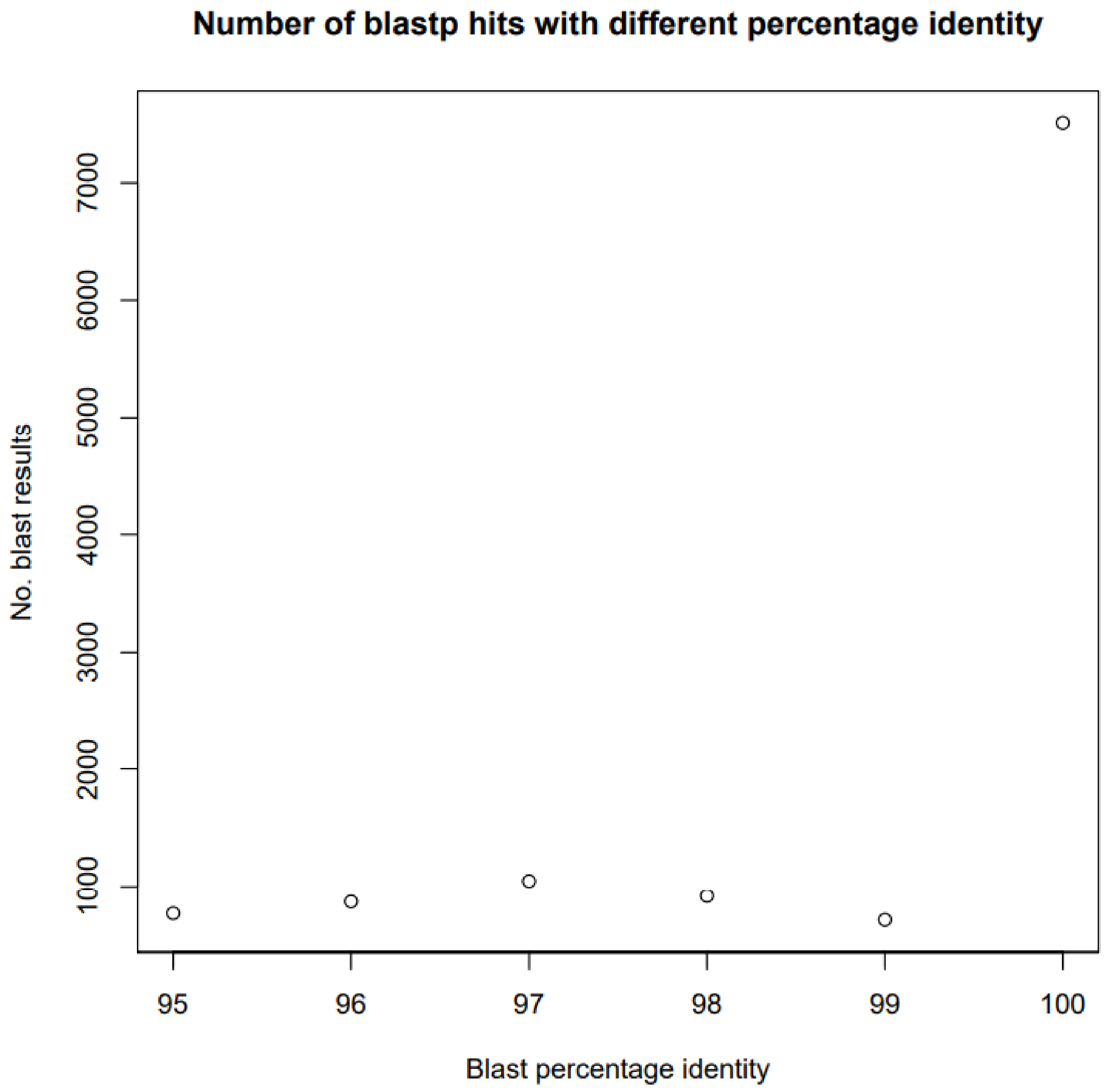

Number of blastp hits with different percentage identity
No. blast results
1000
2000
3000
4000
5000
6000
7000
95
96
97
98
99
100
Blast percentage identity

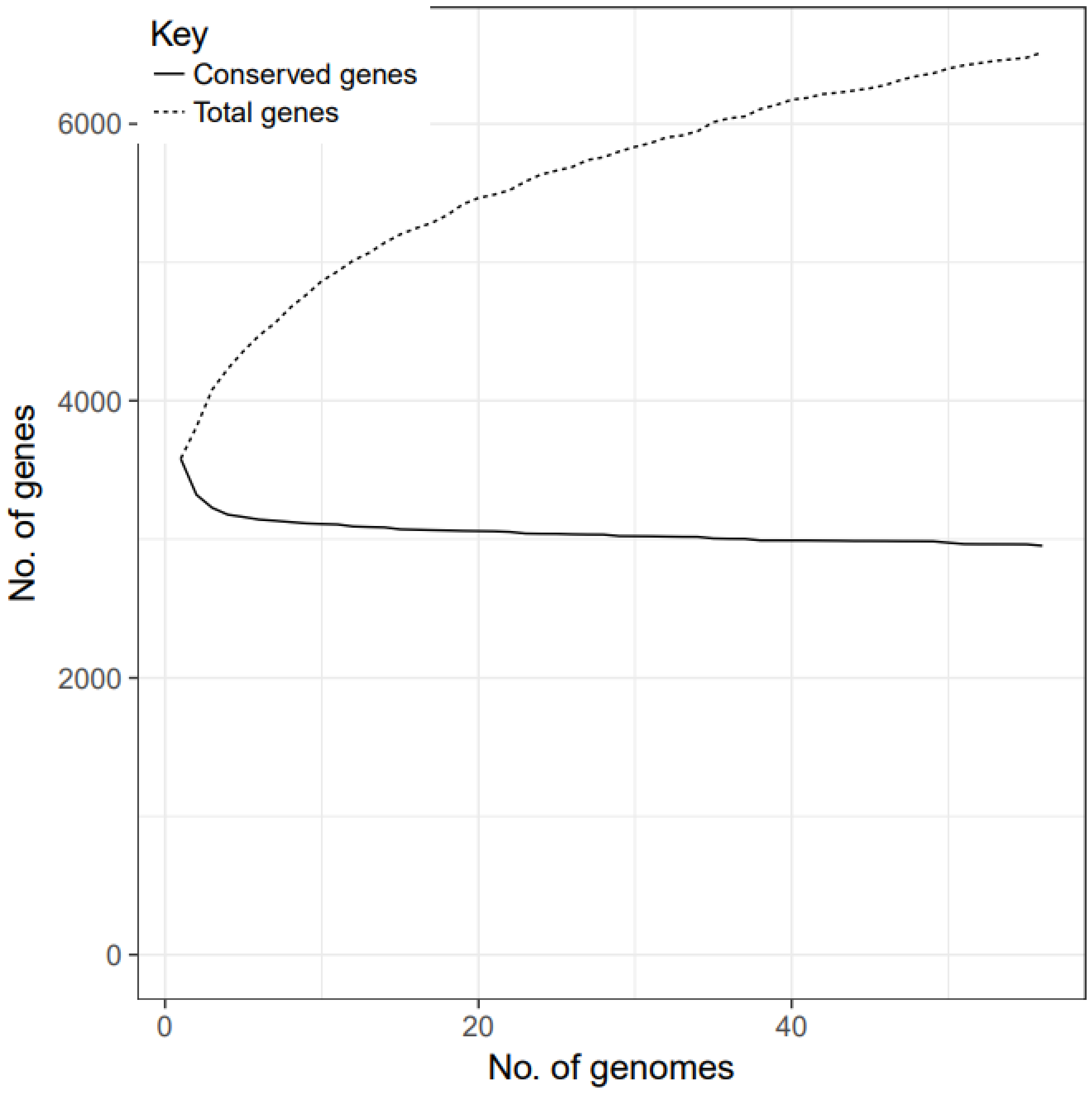
Key
Conserved genes
Total genes
6000
4000
2000
0
0
20
40
No. of genomes
No. of genes

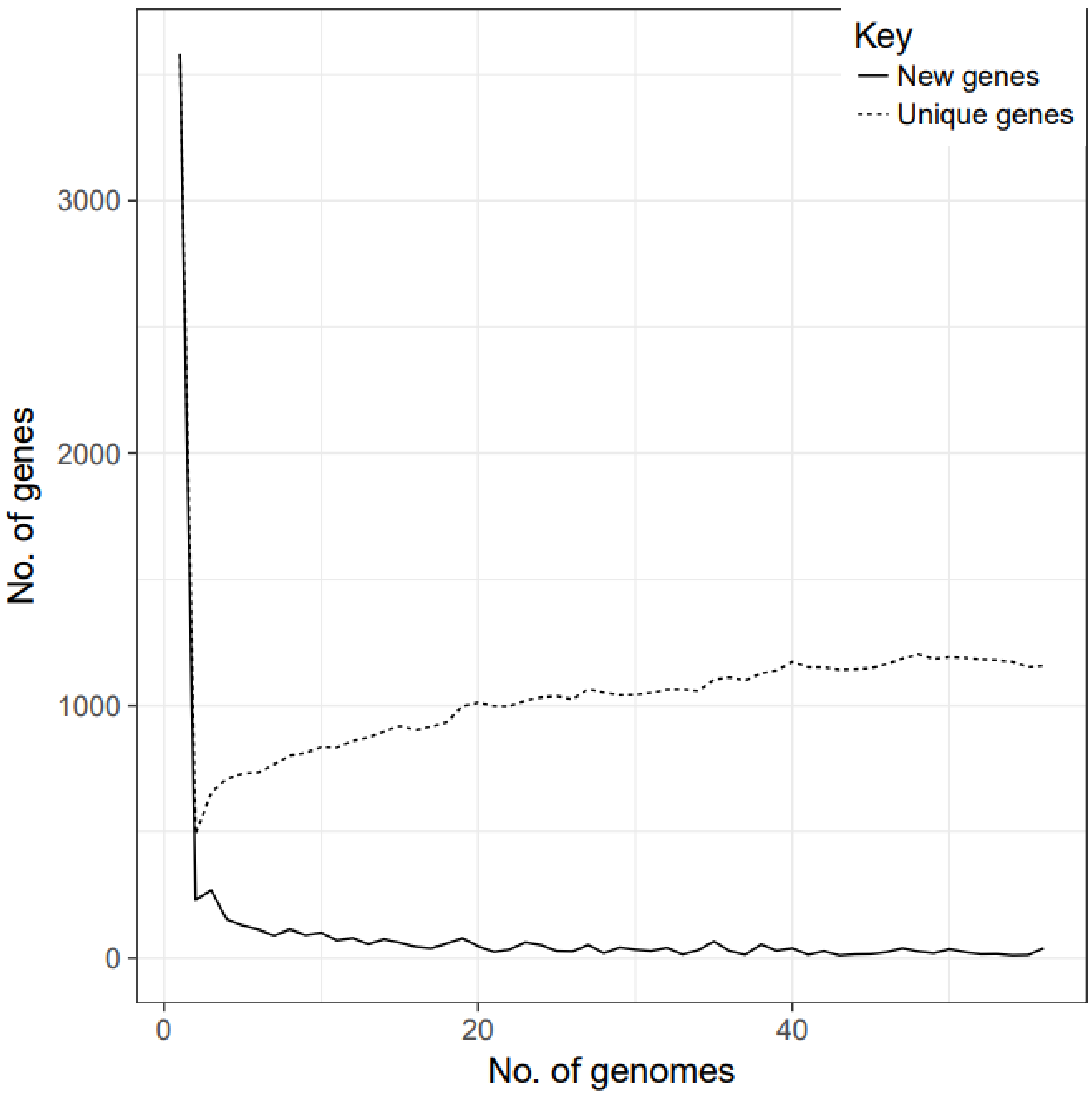

Key
New genes
Unique genes
3000
2000
1000
0
No. of genes
0
20
40
No. of genomes

S2 File: FastGeP matrix in iTOL output file

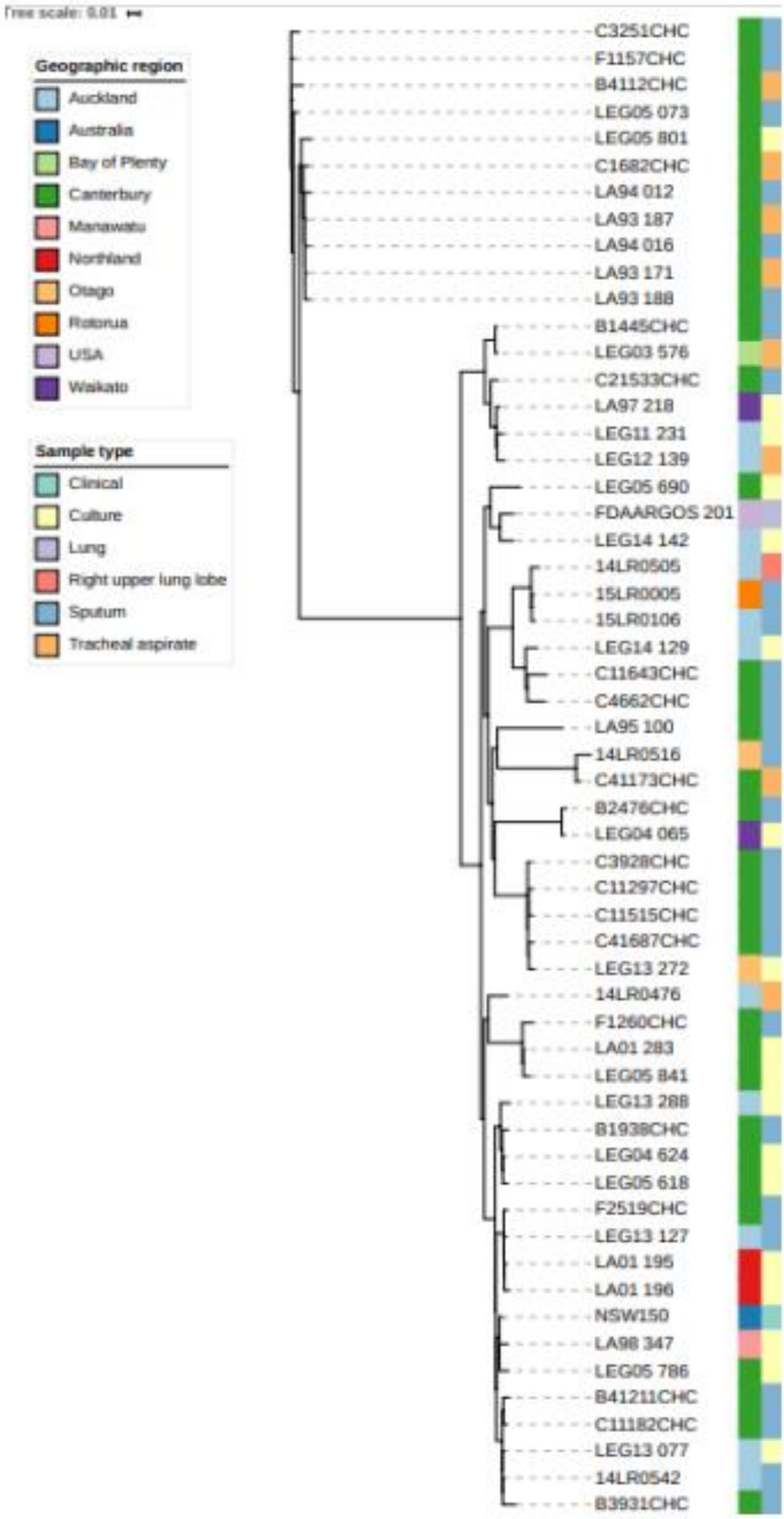

S3 File: Molecular Clock and Tree Model Trialing Model comparison

bModelTest [1] was used to choose the substitution model for the 54 *Legionella longbeachae* isolates, based on 2,338 non-recombinant SNPs padded with 1,306,681 adenine, 765,717 cytosine, 772,189 guanine and 1,298,289 nucleotides 100 million steps. The model with the most posterior support was 121123. This model was formed by connecting substitutions in a Generalise Time Reversible (GTR) model [2].

The 54 *L. longbeachae* isolates phylogenetics were modelled by placing the 2,338 non-recombinant SNPs padded with 1,306,681 adenine, 765,717 cytosine, 772,189 guanine and 1,298,289 nucleotides into BEAST 2.5 [3], and running combinations of molecular clock (strict , random [4] and uncorrelated relaxed [5]) and tree (constant coalescent and Extended Bayesian Skyline [6]) models for 100 million steps. Model combinations with sufficient chain convergence (posterior estimated sample size (ESS) over 200) were compared using Nested Sampling (NS) [7] with 10,000 chains, 5,000 subchains and 10 particles (Table S1). The model combination with the highest Marginal Likelihood Estimate (MLE) was further analysed.

Table 1. ESS and MLE values of model combinations in BEAST

Clock	Tree	ESS (posterior)	NS (MLE)	Standard deviation
Random	Constant	17		
Random	EBS	9		
Relaxed	Constant	490	-5641698.6	5.43
**Relaxed**	**EBS**	**785**	**-5641693.5**	**5.67**
Strict	Constant	1830	-5641747.7	5.29

Chosen model combination is boldened.

References

1. Bouckaert RR, Drummond AJ. bModelTest: Bayesian phylogenetic site model averaging and model comparison. BMC Evol Biol. **2017**; 17(42):1–11.
2. Tavare S. Some probabilistic and statistical problems in the analysis of DNA sequences. Am Math Soc. **1986**; 17:57–86.
3. Bouckaert R, Heled J, Kühnert D, et al. BEAST 2: A software platform for Bayesian evolutionary analysis. PLoS Comput Biol [Internet]. **2014**; 10(4):1–6. Available from: https://www.scopus.com/inward/record.uri?eid=2-s2.0-84901305512&doi=10.1371%2Fjournal.pcbi.1003537&partnerID=40&md5=5515082bdf0d92199f98e1acae7385fb
4. Drummond AJ, Suchard MA. Bayesian random local clocks, or one rate to rule them all. BMC Biol [Internet]. **2010**; 8:1–12. Available from: https://www.scopus.com/inward/record.uri?eid=2-s2.0-77956488734&doi=10.1186%2F1741-7007-8-114&partnerID=40&md5=352795a10a476b4c21172d24ee531622
5. Drummond AJ, Ho SYW, Phillips MJ, Rambaut A. Relaxed phylogenetics and dating with confidence. PLoS Biol [Internet]. **2006**; 4(5):699–710. Available from: https://www.scopus.com/inward/record.uri?eid=2-s2.0-33344465800&partnerID=40&md5=05df21b702e736b9d8eb72c21b2088c3
6. Heled J, Drummond AJ. Bayesian inference of population size history from multiple loci. BMC Evol Biol [Internet]. **2008**; 8(1):1–15. Available from:

https://www.scopus.com/inward/record.uri?eid=2-s2.0-60549111699&partnerID=40&md5=181e2952d1c48734d15ea5675d2f6327
7. Maturana P, Brewer BJ, Klaere S, Bouckaert RR. Model selection and parameter inference in phylogenetics using Nested Sampling. Syst Biol. **2018**; 68(2):219–233

S4 File: Code used to perform statistical analyses and visualisation of the methylome data

Methylome analysis for Slow et al Legionalla longbeachae genome

Background

This docuemtn records the methods and code use to produce the methylome analynsis described in the body of the paper.

As a first step, we define a number of R functions that will be used in further analyses.

```
library(reshape)
library(ggplot2)
library(stringr)
library(ggpubr)


theme_set(theme_pubr())
read_bed <- function(fname){
    res <- read.table(fname, sep="\t", stringsAsFactors=FALSE)
    names(res)[1:3] <- c("chrom", "start", "end")
    res
}

get_locus_tag <- function(x){
    str_match(x, ";locus_tag=(B0B39_\\d+)")[,2]
}

methyl_palette <- c(
 m6A_light = "#a6cee3",
 m6A = "#1f78b4",
 m4C_light = "#b2df8a",
 m4C = "#33a02c"
)

cog_cols <- read.table("../COGcoloursExtra.txt",
                       comment.char="'", sep="\t", header=TRUE)
cog_pal <- structure(cog_cols$InitialHexColorCode,
                     .Names=as.character(cog_cols$COG))
```

Modified bases calculated in genome windows

The portion of modifified "A" and "C" bases was calculated over 1kb windows, using bedtools to calculate both the denominator (number of each base) and the numerate (the number that were modified)

```
bedtools makewindows -w 1000 -g Legionella_both_mods.genome > 1kb_windows.bed
bedtools intersect -c -a 1kb_windows.bed -b m6A.gff > m6A_hist.bed
bedtools intersect -c -a 1kb_windows.bed -b m4C.gff > m4C_hist.bed
bedtools nuc -fi Legionella_both_mods.fa -bed 1kb_windows.bed > nuc_content.tsv
```

Appropriate values for each modification were calculated in R.

```r
nuc_content_windows <- read.table("nuc_content.tsv", stringsAsFactors=FALSE)
nA <- rowSums(nuc_content_windows[,c(6,9)])
nC <- rowSums(nuc_content_windows[,7:8])
m6A <- read_bed("m6A_hist.bed")
m6A$f <- m6A$V4 / nA
m4C <- read_bed("m4C_hist.bed")
m4C$f <- m4C$V4 / nC
```

These values were combiend to visualise the distribution of each modification across the chromosome and plasmid sequence.

```r
both_mods <- data.frame(m4C[,-4], m6A=m6A$f)
names(both_mods)[4] <- "m4C"
molten_mods <- melt(both_mods, id.vars=c("chrom", "start", "end"))
molten_mods$chrom <- factor(molten_mods$chrom, levels=c("CP020894.3", "CP020894.2")

p_distr <- ggplot(molten_mods, aes(start, value, colour=variable)) +
    geom_step() +
    facet_grid(variable~chrom, space="free", scales="free") +
    scale_y_continuous("Porpotion of bases modified") +
    scale_x_continuous("Position (Mb)", label=function(x) x/1e6) +
    scale_colour_manual(values=methyl_palette)

ggsave("figure_elements/Figure_methyl_chrom.svg", p_distr)
```

```
## Saving 6.5 x 4.5 in image
```

```r
ggsave("figure_elements/Figure_methyl_chrom.png", p_distr)
```

```
## Saving 6.5 x 4.5 in image
```

Repeat protein

The most striking result in the above plots is the "spike" in m6A methylation near to the start of the chromosome. To investigate this further we identified genes within this window and its immediate neighbours. First by writing a bed file from R.

```r
most_a <- m6A[which.max(m6A$f),]
most_a$start <- most_a$start - 1000
most_a$end <- most_a$end + 1000
write.table(most_a, row.names=FALSE, col.names=FALSE,
            quote=FALSE, sep="\t", "m6A_spike.bed")
```

Then by isolating overlapping genes with bedtools.

```
bedtools intersect -a CDS.gff  -b m6A_spike.bed  > spike_CDS.gff
```

The previcise location (and strand) of each modification was then plotted, showing the over-representation of modified "A" in a repeat-rich protein.

```r
mod_by_base <- read.table("spike_per_base.gff", sep="\t")
mod_by_base$height <- ifelse(mod_by_base$V6 == "+", .4, -.4)

genes <- read.table("spike_CDS.gff", sep="\t")
genes$LT <- str_match(genes$V9, ";locus_tag=(B0B39_\\d+)")[,2]
```

```r
p_lollypop <- ggplot() +
    geom_hline(yintercept=0) +
    geom_segment(data=mod_by_base, aes(x=V4, xend=V4, y=0, yend=height)) +
    geom_rect(data=genes, aes(xmin=V4, xmax=V5, ymin=-0.15, ymax=0.15),
              fill="white", colour='black') +
    geom_text(data=genes, aes(x=(V4+V5)/2, y=0, label=LT))+
    geom_point(data=mod_by_base, aes(x=V4, y=height),
               fill=methyl_palette['m6A'], size=5, shape=21) +
    scale_x_continuous("Position in chromosome (Mb)", label=function(x) x/1e6) +
    theme(axis.title.y=element_blank(),
        axis.text.y=element_blank(),
        axis.ticks.y=element_blank())

ggsave("figure_elements/lollypops.png", p_lollypop)
```

```
## Saving 6.5 x 4.5 in image
```

```r
ggsave("figure_elements/lollypops.pdf", p_lollypop)
```

```
## Saving 6.5 x 4.5 in image
```

Functional analyses

We tested whether methylated C bases may be associated with protein coding genes, or particular functions of those genes. We first focused on comparing the coding and non-coding portions of the genome. As a first step, we calculated the toal number of "C" bases avaiable to be modified in both coding non non-coding portions of the genome

```
bedtools complement -i CDS.bed -g CP020894_v3.size  > intergenic.bed
bedtools nuc -fi CP020894_v3.fa -bed intergenic.bed | awk '{s+=$8; s+=$7}END{print
bedtools nuc -fi CP020894_v3.fa -bed CDS.bed | awk '{s+=$8; s+=$8}END{print s}'
```

With the total number of bases calculated, we identified the number modified in each genomic component. (cut, sort and unique ensure each base is counted only once).

```
bedtools intersect -a ../m4C.bed  -b intergenic.bed  | cut -f1,2 | sort | uniq | w
bedtools intersect -a ../m4C.bed  -b CDS.bed  | cut -f1,2 | sort | uniq  | wc -l
```

That gives the following numbers.

```r
by_base <- read.csv("CDS_v_intergenic.csv")
p_dynamite <- ggplot(by_base, aes(site_type, p_mod, ymax=upper, ymin=lower)) +
    geom_col(fill=methyl_palette["m4C"], colour='black', size=0.8) +
    geom_errorbar(width=0.3, size=1) +
    xlab("Site type") +
    ylab("Proportion of 'C's modified")

ggsave("figure_elements/dynamite.png", p_dynamite)
```

```
## Saving 6.5 x 4.5 in image
```

```r
ggsave("figure_elements/dynamite.pdf", p_dynamite)
```

```
## Saving 6.5 x 4.5 in image
```

Finally, we looked for any differences between in methylation between genes assigned to difference COGs. Here we plot the proportion of "C"s modified in each gene, shaded by the COG classification assigned to that gene.

```r
meth_by_cog <- read.csv("sup_data/meth_by_COG.csv")
p_cog <- ggplot(meth_by_cog, aes(COG, p_mod, fill=COG)) +
    geom_boxplot(outlier.shape = NA) +  #only plot outliers once
    geom_jitter(width=0.1, height=0, shape=21, colour='black') +
    scale_y_continuous("proportion of 'C's modified") +
    scale_fill_manual(values=cog_pal) +
    theme(legend.position="none")


ggsave("figure_elements/COG_rainbow.png", p_cog)
```

```
## Saving 6.5 x 4.5 in image
```

```r
ggsave("figure_elements/COG_rainbow.pdf", p_cog)
```

```
## Saving 6.5 x 4.5 in image
```
